# Heterogeneous Nuclear Ribonucleoprotein A1 (hnRNP A1) and hnRNP A2 Inhibit Splicing to Human Papillomavirus 16 Splice Site SA409 through a UAG-Containing Sequence in the E7 Coding Region

**DOI:** 10.1128/JVI.01509-20

**Published:** 2020-09-29

**Authors:** Yunji Zheng, Johanna Jönsson, Chengyu Hao, Shirin Shoja Chaghervand, Xiaoxu Cui, Naoko Kajitani, Lijing Gong, Chengjun Wu, Stefan Schwartz

**Affiliations:** a Department of Laboratory Medicine, Lund University, Lund, Sweden; b China Institute of Sport and Health Sciences, Beijing Sport University, Beijing, Haidian District, China; c The First Affiliated Hospital of Shandong First Medical University, Jinan, Shandong Province, China; d Institute of Basic Medicine, Shandong First Medical University & Shandong Academy of Medical Sciences, Jinan, Shandong Province, China; International Centre for Genetic Engineering and Biotechnology

**Keywords:** E6, E7, hnRNP A1, papillomavirus, splicing

## Abstract

Human papillomavirus 16 (HPV16) 5′-splice site SD226 and 3′-splice site SA409 are required for production of the HPV16 E7 mRNAs, whereas unspliced mRNAs produce E6 mRNAs. The E6 and E7 proteins are essential in the HPV16 replication cycle but are also the major HPV16 proteins required for induction and maintenance of malignancy caused by HPV16 infection. Thus, a balanced expression of unspliced and spliced mRNAs is required for production of sufficient quantities of E6 and E7 proteins under physiological and pathophysiological conditions. If splicing becomes too efficient, the levels of unspliced E6 mRNAs will decrease below a threshold level that is no longer able to produce E6 protein quantities high enough to significantly reduce p53 protein levels. Similarly, if splicing becomes too inefficient, the levels of spliced E7 mRNAs will decrease below a threshold level that is no longer able to produce E7 protein quantities high enough to significantly reduce pRb protein levels. To determine how splicing between SD226 and SA409 is regulated, we have investigated how SA409 is controlled by the cellular proteins hnRNP A1 and hnRNP A2, two proteins that have been shown previously to control HPV16 gene expression. We found that hnRNP A1 and A2 interacted directly and specifically with a C-less RNA element located between HPV16 nucleotide positions 594 and 604 downstream of SA409. Overexpression of hnRNP A1 inhibited SA409 and promoted production of unspliced E6 mRNAs at the expense of the E7 mRNAs, whereas overexpression of hnRNP A2 inhibited SA409 to redirect splicing to SA742, a downstream 3′-splice site that is used for generation of HPV16 E6̂E7, E1, and E4 mRNAs. Thus, high levels of either hnRNP A1 or hnRNP A2 inhibited production of the promitotic HPV16 E7 protein. We show that the hnRNP A1 and A2 proteins control the relative levels of the HPV16 unspliced and spliced HPV16 E6 and E7 mRNAs and function as inhibitors of HPV16 E7 expression.

**IMPORTANCE** Human papillomavirus type 16 (HPV16) belongs to the high-risk-group of HPVs and is causing a variety of anogenital cancers and head and neck cancer. The two HPV16 oncoproteins E6 and E7 prevent apoptosis and promote mitosis and are essential for completion of the HPV16 life cycle and for transformation of the infected cell and maintenance of malignancy. E6 and E7 are produced from two mRNAs that are generated in a mutually exclusive manner by alternative splicing. While E6 protein is made from the unspliced mRNA, E7 is made from the spliced version of the same pre-mRNA. Since sufficient quantities of both E6 and E7 are required for malignant transformation, this intricate arrangement of gene expression renders E6 and E7 expression vulnerable to external interference. Since antiviral drugs to HPV16 are not available, a detailed knowledge of the regulation of HPV16 E6 and E7 mRNA splicing may uncover novel targets for therapy.

## Abstract (precis)

Human papillomavirus type 16 (HPV16) belongs to the high-risk-group of HPVs and is causing a variety of anogenital cancers and head and neck cancer. The two HPV16 oncoproteins E6 and E7 prevent apoptosis and promote mitosis and are essential for completion of the HPV16 life cycle and for transformation of the infected cell and maintenance of malignancy. E6 and E7 are produced from two mRNAs that are generated in a mutually exclusive manner by alternative splicing. While E6 protein is made from the unspliced mRNA, E7 is made from the spliced version of the same pre-mRNA. Since sufficient quantities of both E6 and E7 are required for malignant transformation, this intricate arrangement of gene expression renders E6 and E7 expression vulnerable to external interference. Since antiviral drugs to HPV16 are not available, a detailed knowledge of the regulation of HPV16 E6 and E7 mRNA splicing may uncover novel targets for therapy.

## INTRODUCTION

Human papillomaviruses (HPV) are small DNA viruses with a strict tropism for human epithelial cells. A subset of the HPVs is mainly sexually transmitted and has tropism for mucosal cells. The vast majority of these HPV infections are asymptomatic, and the infections are cleared by the immune system of the host. However, the high-risk HPVs can establish chronic persistent infections that last for many years or decades (1, 2). These infections may give rise to cervical lesions that progress to cervical cancer (3). More than 99% of all cervical cancers contain HPV DNA, and epidemiological studies have established that the most common high-risk HPV is HPV16 and that HPV16 is present in approximately 50% of all cervical cancers (4). HPV16 encodes at least eight distinct protein-expressing genes. Among those, the E6 and E7 genes are of particular interest. They not only carry out essential functions in the HPV16 replication cycle but also contribute to induction of cervical lesions as well as induction and maintenance of cervical cancer (5–7). Continued expression of HPV16 E6 and E7 is required for survival and proliferation of HPV16-driven cancer cells (8). This requirement is mainly attributed to the ability of HPV16 E7 to drive cell proliferation through its association with pRB and the liberation of E2F (9), while E6 targets p53 for degradation, thereby preventing apoptosis (10). However, the roles of E6 and E7 in carcinogenesis may be more complex than that, since both E6 and E7 interact with additional proteins.

**FIG 1:**
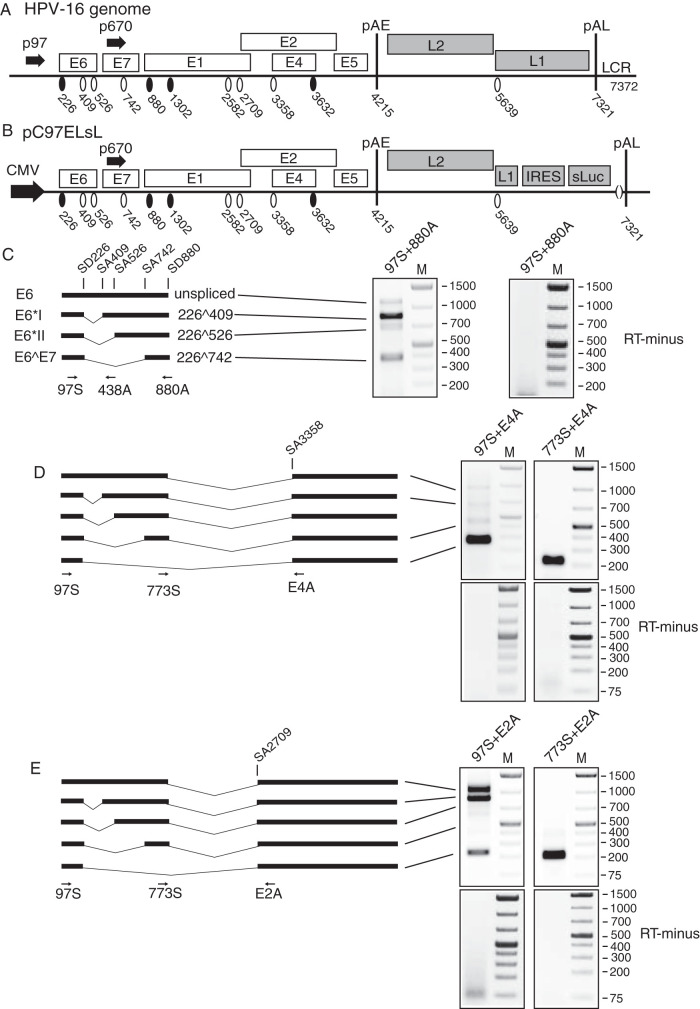
Alternatively spliced HPV16 mRNAs produced by subgenomic HPV16 expression plasmid pC97ELsL. (A and B) Schematic representation of the HPV16 genome (A) and the HPV16 subgenomic pC97ELsL reporter plasmid (62) (B). Transcription of the HPV16 sequences in the pC97ELsL plasmid is driven by the human cytomegalovirus immediate-early promoter (CMV). HPV16 splice sites are indicated. Numbers refer to the HPV16 reference strain HPV16R (45). Early and late polyadenylation signals pAE and pAL are indicated. p97 and p670 indicate HPV16 early and late promoters, respectively. IRES, internal ribosome entry site; sluc, secreted luciferase. (C, left) Schematic representation of a subset of alternatively spliced mRNAs produced by pC97ELsL and amplified by RT-PCR primers 97S and 880A or 438A. Arrows indicate the location of RT-PCR primers. The names of the first open reading frame (orf) on each alternatively spliced mRNA are indicated to the left. (Right) RT-PCR with primers 97S and 880A on RNA extracted from HeLa cells transfected with pC97ELsL. Control PCR in the absence of reverse transcriptase (RT-minus) is shown. (D, left) Schematic representation of a subset of alternatively spliced mRNAs produced by pC97ELsL. This subset of HPV16 mRNAs all use HPV16 3′-splice site SA3358 and the HPV16 early polyadenylation signal pAE. Arrows indicate the location of RT-PCR primers 97S, 773S, and E4A. (Right) RT-PCR with indicated primers on RNA extracted from HeLa cells transfected with pC97ELsL. Control PCRs in the absence of reverse transcriptase (RT-minus) are shown. (E, left) Schematic representation of a subset of alternatively spliced mRNAs produced by pC97ELsL. This subset of HPV16 mRNAs all use the HPV16 3′-splice site SA2709 and the HPV16 early polyadenylation signal pAE. Arrows indicate the location of RT-PCR primers 97S, 773S, and E2A. (Right) RT-PCR with indicated primers on RNA extracted from HeLa cells transfected with pC97ELsL. Control PCRs in the absence of reverse transcriptase (RT-minus) are shown.

Transcriptional regulation is important in the control of HPV16 gene expression (11–13). HPV16 makes extensive use of alternative mRNA splicing to produce mRNAs encoding each of the virally encoded proteins (14–18). The various splice sites on the HPV16 mRNAs are controlled by *cis*-acting RNA elements that interact with transacting cellular factors produced by the HPV16-infected cells. Splicing regulatory RNA elements in papillomaviruses were first discovered in bovine papillomaviruses (19–21). As a matter of fact, the HPV16 infection itself alters expression levels of cellular RNA binding proteins that control mRNA splicing, presumably to optimize intracellular conditions for the HPV16 gene expression program (22, 23). Furthermore, HPV16 infection activates the DNA damage response machinery (DDR) (6), and DDR factors recruit RNA binding proteins that modulate HPV16 mRNA splicing and polyadenylation (24, 25). The HPV16 E6 and E7 proteins are produced from two mRNAs that are generated in a mutually exclusive manner: the unspliced mRNAs encode the full-length E6 protein, and the spliced mRNAs encode E7 (26, 27). The splice sites utilized to produce the E7 mRNAs are located within the E6 open reading frame (orf) and are named SD226 (5′-splice site) and SA409 and SA526 (3′-splice site) (Fig. 1A to C) (14, 16, 17). The major and most abundant HPV16 mRNA produced in HPV16-infected cells as well as in HPV16-driven cancers is spliced from SD226 to SA409 (28–31). This splicing event shortens the E6 orf to generate the E6*I-orf located upstream of the E7 orf on this mRNA (32). As a result of the shortened E6 orf and its poor ATG, ribosomes may reinitiate translation at the E7 ATG (26, 33, 34) or reach the downstream E7 orf by leaky scanning (35). Alternative ideas have been presented using less stringent experimental systems (36, 37). In contrast, translation of the E7 orf does not seem to occur on mRNAs that contain an intact, full-length E6 orf upstream of E7 (26, 36). The SD226-SA409 mRNA is specific for high-risk, cancer-associated HPV types and appears to be the major mRNA encoding the promitotic E7 protein. However, splicing to SA409 is not allowed to be too efficient, as that would prevent production of full-length E6 mRNAs. Since the utilization efficiency of SA409 determines the relative levels of the HPV16 E6 and E7 mRNAs, the control of splice site SA409 is of particular interest. We speculate that the intracellular concentrations of RNA binding proteins that control HPV16 E6 and E7 mRNA splicing is of paramount importance for a balanced expression of E6 and E7 mRNAs. Therefore, it is of interest to identify HPV16 RNA elements and cellular RNA binding proteins that control HPV16 E6 and E7 mRNA splicing. The cellular RNA binding proteins hnRNP A1 and hnRNP A2 have previously been shown to regulate HPV16 and HPV18 mRNA splicing. More specifically, hnRNP A1 and A2 have been shown to affect HPV16 E6 and E7 mRNA splicing in an ERK-dependent manner (38), hnRNP A1 have been shown to regulate HPV18 E6 and E7 mRNA splicing (39), and hnRNP A1 and hnRNP A2 control HPV16 late gene expression (40–44). hnRNP A1 is upregulated in HPV16-infected cells, in particular in high-grade cervical lesions as well as cervical cancer (22). Furthermore, expression levels of hnRNP A1 are affected by HPV16 infection. Here, we show that hnRNP A1 and A2 interact with a C-less RNA element in the E7 coding region, thereby inhibiting HPV16 E7 mRNA splicing and HPV16 E7 production. Collectively, these results demonstrate that hnRNP A1 and A2 are major regulators of HPV16 mRNA splicing and HPV16 gene expression.

## RESULTS

### Inhibition of HPV16 3′-splice site SA409 by hnRNP A1 results in production of unspliced E6 mRNAs, whereas inhibition of SA409 by hnRNP A2 results in splicing to alternative 3′-splice site SA742.

The hnRNP A1 and A2 proteins were shown previously to be involved in the regulation of HPV16 gene expression at the level of RNA processing (38–44). To further investigate the effects of hnRNP A1 and hnRNP A2 on HPV16 mRNA splicing, we wished to determine the effect of hnRNP A1 on the splicing of HPV16 mRNAs produced by the HPV16 subgenomic reporter plasmid pC97ELsL (Fig. 1A and B). This plasmid was transfected into HeLa cells, and RNA was extracted and subjected to reverse transcription-PCR (RT-PCR). The various alternatively spliced HPV16 mRNAs produced by pC97ELsL were monitored by RT-PCR using various primer pairs (Fig. 1C to E). The RT-PCR gels in Fig. 1C to E show alternatively spliced HPV16 mRNAs produced in HeLa cells transfected with pC97ELsL. In addition to identifying the mRNAs produced by pC97ELsL, the results also revealed that mRNAs utilizing HPV16 SD226 are efficiently spliced to SA3358 (Fig. 1D), whereas mRNAs utilizing SD880 are more efficiently spliced to SA2709 (Fig. 1E). Absence of detectable plasmid DNA contamination of the RNA samples was confirmed by RT-PCR experiments performed in the absence of reverse transcriptase for all HPV16 primer pairs (RT-minus) (Fig. 1C to E). All RT-PCR products were sequenced to determine their identity. These results identify the HPV16 mRNAs detected by the various RT-PCR primers.

**FIG 2:**
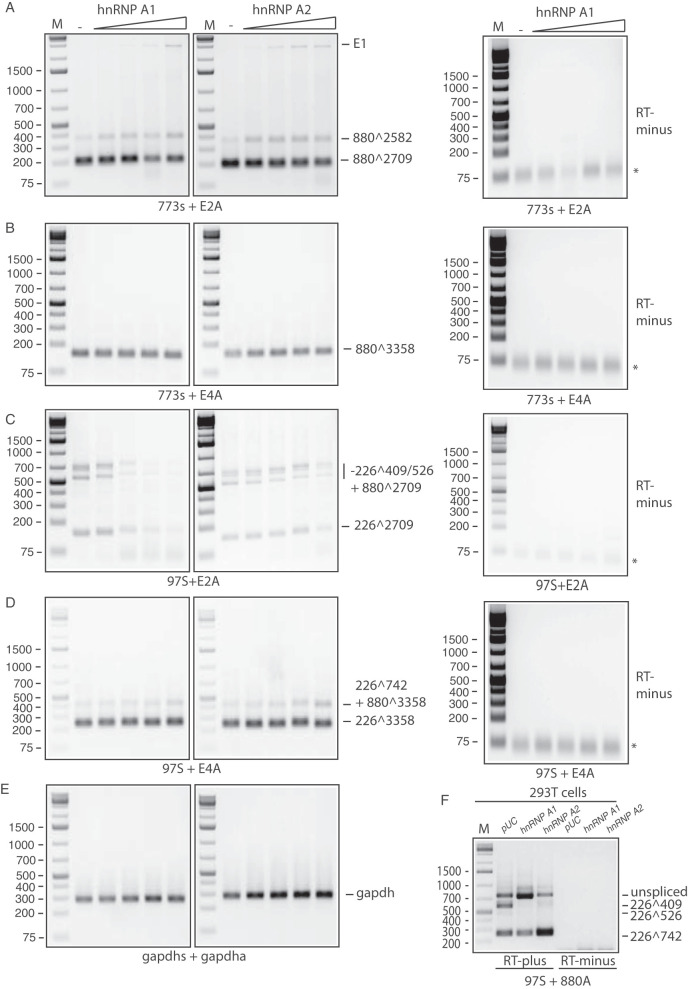
hnRNP A1 and A2 affect splicing between HPV16 5′-splice site SD226 and 3′-splice sites SA409 and SA526. (A to E) RT-PCR on RNA extracted from HeLa cells transfected with pC97ELsL (62) in the absence (−) or presence of 2-fold serially diluted plasmid phnRNP A1 or phnRNP A2. RT-PCR primers are indicated below each gel pair. Control PCRs in the absence of reverse transcriptase (RT-minus) are shown to the left. The HPV16 splice sites used to generate the mRNAs amplified by the RT-PCR primers are indicated to the right. (F) RT-PCR on RNA extracted from 293T cells transfected with pC97ELsL in the absence (−) or presence of plasmid phnRNP A1 or phnRNP A2. RT-PCR primers 97S and 880A were used. The HPV16 splice sites used to generate the mRNAs amplified by the RT-PCR primers are indicated to the right. M, molecular size marker; *, primers.

To monitor the effect of hnRNP A1 or hnRNP A2 on the various alternatively spliced HPV16 mRNAs, serially diluted expression plasmids for hnRNP A1 or hnRNP A2 were cotransfected with HPV16 reporter plasmid pC97ELsL into HeLa cells. First, the mRNAs spliced from HPV16 5′-splice site SD880 to either SA2709 or SA3358 were monitored by RT-PCR. As can be seen from Fig. 2A, B, D, and E, mRNAs spliced from SD880 to either SA2709 or SA3358 were largely unaffected by hnRNP A1 or A2 overexpression. In contrast, mRNAs spliced from SD226 and analyzed with primers 97S and E2AS were strongly reduced by hnRNP A1 but only to a minor extent by hnRNP A2 (Fig. 2C). Absence of detectable plasmid DNA contamination of the RNA samples was confirmed by RT-PCR experiments performed in the absence of reverse transcriptase for all HPV16 primer pairs (RT-minus) (Fig. 2A to D). Since many of the mRNAs detected by primers 97S and E2AS contain exons from the E6 and E7 region (that is, mRNAs spliced from SD226 to either SA409, SA526, or SA742), the results suggested that hnRNP A1 and, to a lesser extent, hnRNP A2 affected splice site SD226, SA409, SA526, or SA742 in the E6 and E7 coding region.

**FIG 3:**
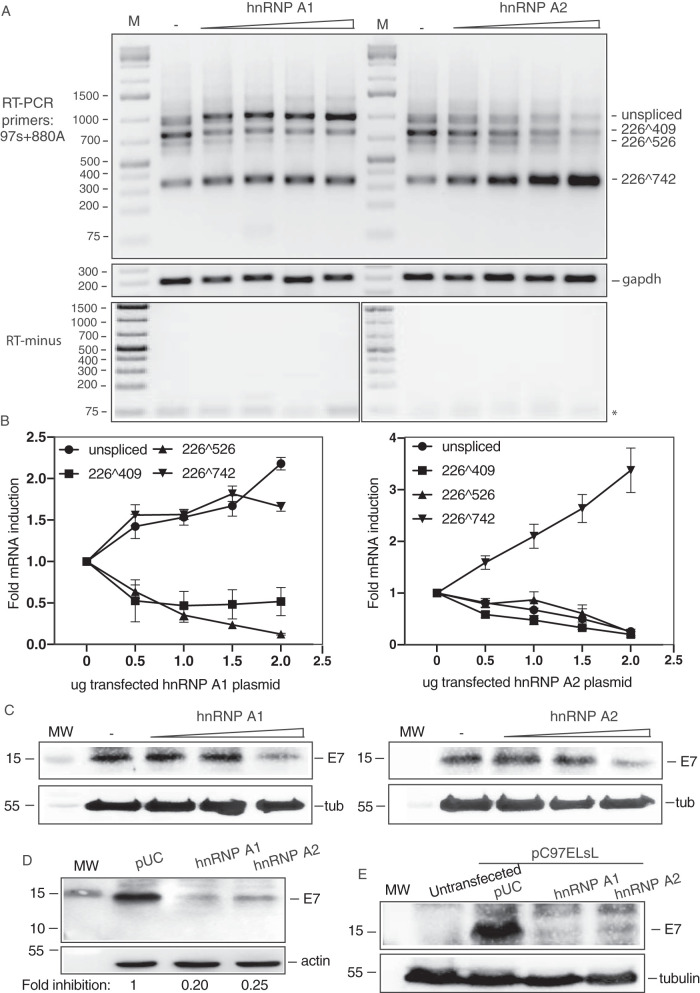
Differential effects of hnRNP A1 and A2 on HPV16 E6 and E7 mRNA splicing. (A) RT-PCR on RNA extracted from HeLa cells transfected with pC97ELsL (62) in the absence (−) or presence of 2-fold serially diluted phnRNP A1 or phnRNP A2. RT-PCR primers are indicated to the left of the gel pair. The HPV16 splice sites used to generate the mRNAs amplified by the RT-PCR primers are indicated to the right. Transfections were performed in triplicates, and representative gel pictures are displayed. Control PCRs in the absence of reverse transcriptase (RT-minus) are shown below RT-PCR gels. M, molecular size marker; *, primers. (B) Densitometric quantification of RT-PCR bands was performed on triplicate transfections. Mean values and standard deviations are shown. The quantitations are displayed as fold difference of HPV16 RT-PCR band intensity obtained with RNA extracted from cells transfected with pC97ELsL in the presence of phnRNP A1 or A2 over band intensities in cells transfected with pC97ELsL in the presence of empty pUC plasmid (−). Band intensity ratios are plotted against micrograms of transfected phnRNP A1 or phnRNP A2 plasmid DNA. (C) Western blotting on cell extracts from HeLa cells transfected with pC97ELsL (0.5 μg) and 2-fold serially diluted phnRNP A1 or phnRNP A2 plasmid (1.5 μg, 0.75 μg, 0.38 μg, and 0.19 μg). Cells were harvested 20 h posttransfection. Blots were stained with antibody specific for HPV16 E7 and actin as described in Materials and Methods. (D) Western blotting on cell extracts from HeLa cells transfected with pC97ELsL (0.5 μg) and 1.5 μg of phnRNP A1 or phnRNP A2 plasmid. The E7 and actin bands were quantified, and fold reduction of E7 protein by hnRNP A1 or hnRNP A2 overexpression is indicated below gel pictures. (E) Western blotting on cell extracts from HeLa cells transfected with pC97ELsL (0.5 μg) and 1.5 μg of phnRNP A1 or phnRNP A2 plasmid. Cell extract from untransfected cells is included. MW, molecular weight marker.

Our results indicated that hnRNP A1 and A2 affected splice sites in the E6 and E7 coding regions, which is in line with previously published results on HPV16 (38) and HPV18 (39). Next, we analyzed the effect of hnRNP A1 and A2 on the HPV16 splice sites SD226, SA409, SA526, and/or SA742 in the E6 and E7 coding region using primer pair 97S and 880A that specifically covers this area (Fig. 1C depicts the structures of the E6 and E7 mRNAs and the location of these primer pairs). Overexpression of hnRNP A1 inhibited splicing to splice sites SA409 and SA526 and resulted in a significant increase in unspliced mRNAs encoding the full-length E6 open reading frame (Fig. 3A and B). Levels of mRNAs spliced from SD226 to SA742 (E6̂E7 mRNAs) also increased at the expense of mRNAs spliced to SA409 and SA526 (Fig. 3A and B). Thus, high levels of hnRNP A1 favored unspliced E6 mRNAs and mRNAs spliced SD226 to SA742 over mRNAs spliced to SA409 and SA526. Overexpression of hnRNP A2 also inhibited production of mRNAs spliced to SA409 and SA526, but, in contrast to hnRNP A1 overexpression, hnRNP A2 overexpression did not promote production of unspliced E6 mRNAs but strongly enhanced alternative splicing from SD226 to SA742 (Fig. 3A and B). This occurred at the expense of the unspliced E6 mRNAs as well as mRNAs spliced to SA409 and SA526 encoding E6*I and E6*II (Fig. 3A and B). Similar results were obtained after transfection of 293T cells (Fig. 2F). Unspliced mRNAs are known to produce primarily E6 protein, while mRNAs spliced to SA409 produce primarily E7 protein, although they are also predicted to produce the E6*I protein, and mRNAs spliced to SA742 produce the E6̂E7 protein. As predicted, overexpression of either hnRNP A1 or hnRNP A2 reduced E7 protein production (Fig. 3C). There was a 4- to 5-fold reduction in E7 protein levels (Fig. 3D). The E7 antibody specifically detected the HPV16 E7 protein in pC97ELsL-transfected cells and not in untransfected cells (Fig. 3E). As an additional control for the E7 antibody specificity, we transfected HeLa cells with plasmid pE6E7F, from which Flag-tagged E7 is produced and stained with E7 antibody. High levels of E7 protein were observed in transfected cells, whereas E7 protein was undetectable in untransfected cells (data not shown), further supporting the specificity of the antibody. Although hnRNP A1 is predicted to enhance E6 production, we were unable to detect the E6 protein (data not shown). We concluded that hnRNP A1 and A2 both inhibited splicing to SA409 and SA526 and thereby inhibited production of E7 protein, but the alternative splicing outcome of this inhibition differed between the two proteins hnRNP A1 and hnRNP A2.

### Differential effect of hnRNP A1 and hnRNP A2 on alternative splicing of the HPV16 L1 mRNAs.

We have previously shown that hnRNP A1 and A2 bind to splicing silencers on the HPV16 L1 mRNAs (40–43). Therefore, we investigated if overexpression of hnRNP A1 or A2 affected alternative splicing of HPV16 L1 mRNAs produced from pC97ELsL. Although the majority of HPV16 L1- and L2-encoding mRNAs are produced from the HPV16 late promoter, HPV16 L1- and L2-encoding mRNAs initiated at the early promoter also have been identified in HPV16-infected cells (45). Analyses of the low levels of L1 mRNAs produced by pC97ELsL were performed with primers 97S and L1A or 773S and L1A (data not shown). The results revealed that overexpression of hnRNP A1 inhibited inclusion of the central exon located between SA3358 and SD3632 on the HPV16 L1 mRNAs and favored production of L1 mRNAs spliced from either SD226 or SD880 directly to SA5639 (data not shown). In contrast, overexpression of hnRNP A2 favored inclusion of the central exon between SA3358 and SD3632 on all L1 mRNAs detected with primers 97S and L1A or 773S and L1A (data not shown). Therefore, these results support the idea that hnRNP A1 and A2 differ in their effects on HPV16 mRNA splicing.

### hnRNP A1 acts on splicing inhibitory RNA sequences upstream of HPV16 nucleotide position 604 in the HPV16 E7 coding region.

**FIG 4:**
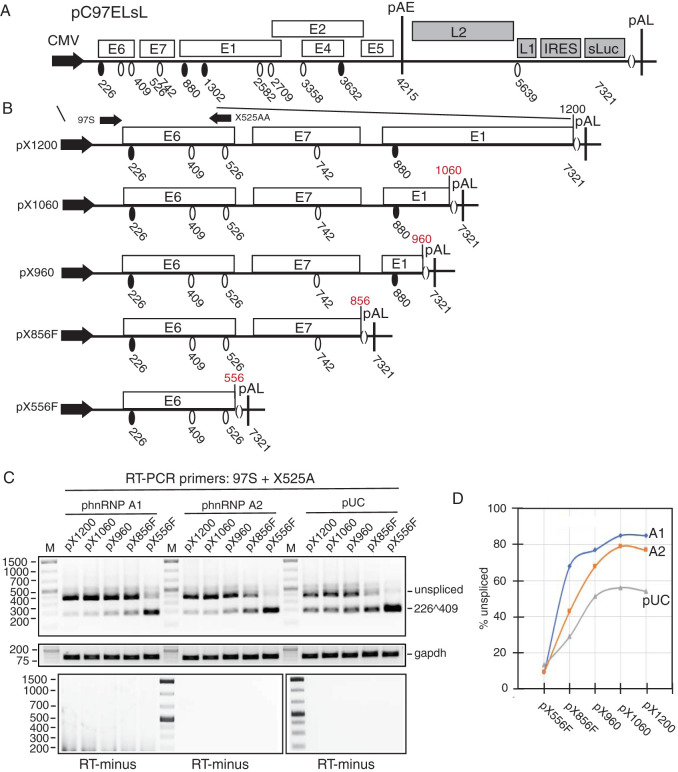
hnRNP A1 and A2 target HPV16 sequences located between HPV16 genomic nucleotide positions 556 and 856. (A) Schematic representation of the HPV16 subgenomic pC97ELsL reporter plasmid (62). Transcription of the HPV16 sequences in the pC97ELsL plasmid is driven by the human cytomegalovirus immediate-early promoter (CMV). HPV16 splice sites are indicated. Numbers refer to the HPV16 reference strain HPV16R (45). Early and late polyadenylation signals pAE and pAL are indicated. IRES, internal ribosome entry site; sluc, secreted luciferase. (B) Schematic representation of HPV16 subgenomic reporter plasmids pX1200, pX1060, pX960, pX856F, and pX556F. Transcription of the HPV16 sequences is driven by the human cytomegalovirus immediate-early promoter (CMV). RT-PCR primers 97S and X525A are indicated. HPV16 sequences in the deletion mutants pX1200, pX1060, pX960, pX856F, and pX556F end at HPV16 nucleotide positions 1200, 1060, 960, 856, and 556. HPV16 splice sites are indicated. Nucleotide positions refer to the nucleotide position of the HPV16 reference genome HPV16R. (C) RT-PCR on RNA extracted from HeLa cells transfected with plasmids pX1200, pX1060, pX960, pX856F, or pX556F in the absence (−) or presence of phnRNP A1 or phnRNP A2. The bands representing unspliced HPV16 E6 mRNAs and HPV16 mRNAs spliced from SD226 to SA409 are indicated to the right. cDNAs were amplified by RT-PCR with primers 97S and X525A. RT-minus, RT-PCR performed in the absence of reverse transcriptase. (D) Quantitations were performed on triplicates. Percent unspliced HPV16 mRNA is calculated for each HPV16 plasmid transfected with either pUC plasmid or hnRNP A1 or hnRNP A2 expression plasmid and plotted against the transfected HPV16 expression plasmid.

Having established that high levels of hnRNP A1 and A2 inhibited splicing between HPV16 splice sites SD226 and SA409, we wished to the map the target site for hnRNP A1 and hnRNP A2 on the HPV16 E6 and E7 mRNAs. To this end, we generated a number of subgenomic HPV16 expression plasmids from pC97ELsL (Fig. 4A and B). Plasmids pX1200, pX1060, pX960, pX856F, and pX556F were transfected into HeLa cells in the presence of empty pUC plasmid (−) or plasmids encoding hnRNP A1 or hnRNP A2, RNA was extracted, and RT-PCR was performed with primers 97S and 438A (Fig. 4C). The numerical digits in the plasmid names represent the endpoint of each deletion and refer to nucleotide position in the HPV16R sequence. Analysis of RT-PCR products from the deletion mutants cotransfected with empty pUC plasmid revealed that mRNAs produced from pX556F were efficiently spliced between SD226 and SA409 (Fig. 4C), whereas HPV16 mRNAs produced from the longer plasmids pX1200, pX1060, pX960, and pX856F were less efficiently spliced (Fig. 4C). Quantitation of the bands and plotting percent unspliced versus the endpoint in the HPV16 genome of the various plasmids revealed that only around 10% of the mRNAs produced by the smallest plasmid, pX556F, were unspliced when cotransfected with empty pUC plasmid (Fig. 4D). Percent unspliced mRNA increased when downstream sequences were included, and it was around 30% with plasmid pX856F and peaked at around 55% for plasmids pX960, pX1060, and pX1200 (Fig. 4C and D), indicating that multiple splicing inhibitory RNA sequences were present downstream of HPV16 nucleotide position 556. RT-PCR bands were undetectable in control PCR experiments performed on the same RNA samples in the absence of reverse transcriptase (data not shown). These splicing inhibitory RNA sequences may be the target sequences for hnRNP A1 and A2.

To determine how hnRNP A1 and hnRNP A2 affected splicing of mRNAs produced from the various HPV16 deletion mutants pX1200, pX1060, pX960, pX856F, and pX556F (Fig. 4B), RNA was extracted from HeLa cells transfected with these plasmids in the presence of hnRNP A1 or hnRNP A2 and analyzed by RT-PCR with primers 97S and 438A (Fig. 4B). The results revealed that overexpression of hnRNP A1 and A2 did not inhibit splicing from plasmid pX556F (Fig. 4C and D), demonstrating that the target sequences for hnRNP A1 and A2 were absent from pX556F. In contrast, overexpression of hnRNP A1 and A2 efficiently inhibited splicing of mRNAs produced from plasmids pX1200, pX1060, pX960, and pX856F (Fig. 4C and D). These results suggested that overexpression of hnRNP A1 and A2 (hnRNP A1 in particular) acted through splicing inhibitory sequences located downstream of HPV16 nucleotide position 556.

**FIG 5:**
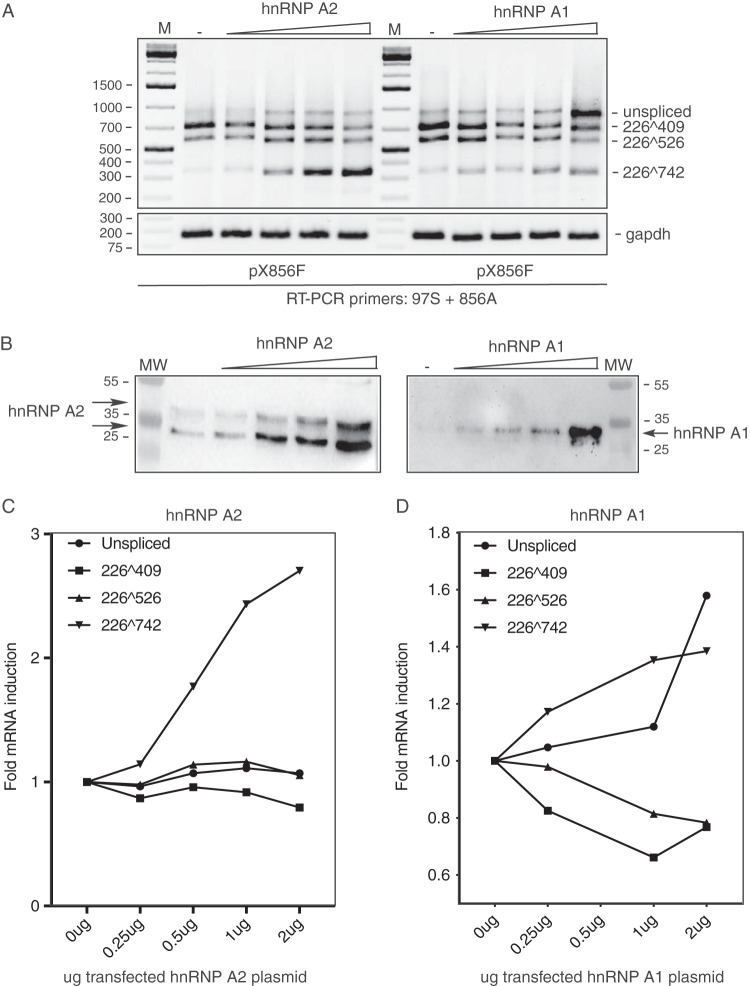
Dose-dependent, differential effects of hnRNP A1 and A on HPV16 E6 and E7 mRNA alternative splicing. (A) RT-PCR on RNA extracted from HeLa cells transfected with pX856F in the absence (−) or presence of 2-fold serially diluted phnRNP A1 or phnRNP A2 (2, 1, 0.5, and 0.25 μg). RT-PCR primers were 97S and 856A. The splicing pattern each RT-PCR band represents is indicated to the right. M, molecular size marker. (B) Western blotting on cell extracts from HeLa cells transfected with serially diluted phnRNP A1 or phnRNP A2 plasmid. Blots were stained with antibodies specific for hnRNP A1 or A2 as described in Materials and Methods. MW, molecular weight marker. (C) Densitometric quantification of the RT-PCR bands in panel A. The quantitations are displayed as fold difference of HPV16 RT-PCR band intensity obtained with RNA extracted from HeLa cells transfected with phnRNP A1 or phnRNP A2 plasmid over band intensities in cells transfected with empty pUC plasmid. Fold difference is plotted against the amount of transfected phnRNP A1 or phnRNP A2 plasmid.

To provide further support for the results that indicated that both hnRNP A1 and A2 inhibited HPV16 mRNA splicing from HPV16 plasmid pX856F (the smallest HPV16 plasmid that responded to hnRNP A1 and A2), serial dilutions of hnRNP A1 or A2 plasmid were cotransfected with pX856F, RNA was extracted, and RT-PCR was performed. The results revealed that hnRNP A1 and A2 both inhibited splicing between SD226 and SA409 in a dose-dependent manner (Fig. 5A and B). RT-PCR bands were undetectable in control PCR experiments performed on the same RNA samples in the absence of reverse transcriptase (data not shown). As expected, hnRNP A1 promoted production of the unspliced HPV16 E6 mRNA (Fig. 5A and D), whereas hnRNP A2 promoted production of HPV16 mRNAs spliced from SD226 to SA742 (Fig. 5A and C). In conclusion, both hnRNP A1 and A2 acted on HPV16 sequences located upstream of HPV16 nucleotide position 856 to inhibit splicing between SD226 and SA409. However, the outcome of this splicing inhibition differed between hnRNP A1 and A2.

**FIG 6:**
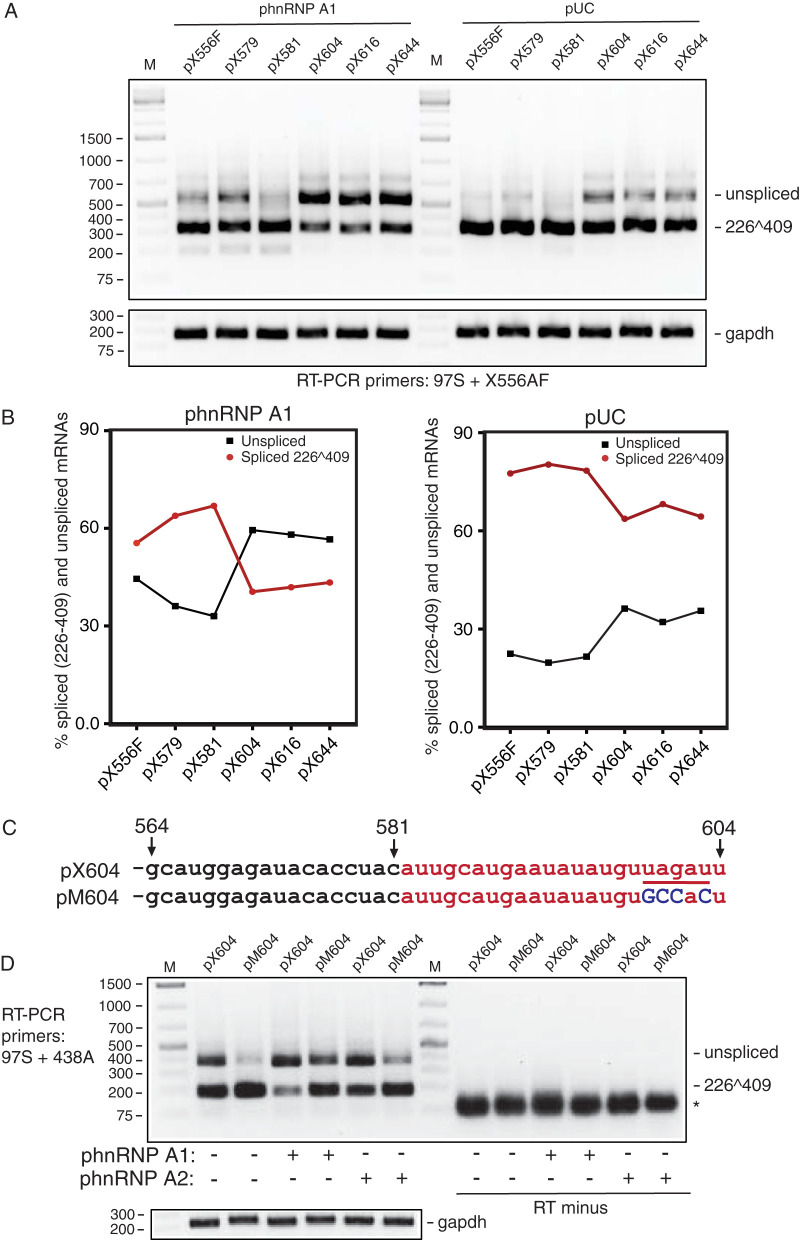
Mapping of the hnRNP A1 target site in the HPV16 E7 coding region to a splicing-inhibitory HPV16 RNA sequence located between HPV16 genomic nucleotide positions 579 and 604. (A) RT-PCR on RNA extracted from HeLa cells transfected with plasmid pX556F, pX579, pX581, pX604, pX616, or pX644 in the absence (pUC) or presence of phnRNP A1. The bands representing unspliced HPV16 E6 mRNAs and HPV16 mRNAs spliced from SD226 to SA409 are indicated to the right. cDNAs were amplified by RT-PCR primers 97S and X556AF. (B) Quantitation of results in panel A. The two lines represent percentage unspliced or spliced HPV16 mRNA calculated for each HPV16 plasmid transfected with either pUC plasmid or hnRNP A1 expression plasmid and plotted against each transfected HPV16 expression plasmid. (C, upper) The wild-type HPV16 sequence from HPV16 nucleotide positions 564 to 604 is displayed. The region between HPV16 nucleotide positions 581 and 604 that mediates splicing inhibition is marked in red. The UAG-containing sequence that constitutes a potential target site for hnRNP A1 and A2 is underlined. This sequence represents the sequence in wild-type plasmid pX604. (Lower) The mutant HPV16 sequence in which four nucleotide substitutions were introduced is displayed. Nucleotide substitutions are in dark blue and capitalized. This sequence represents the sequence in mutant plasmid pM604. (D) RT-PCR on RNA extracted from HeLa cells transfected with plasmid pX604 or pM604 in the absence (pUC) or presence of plasmids expressing phnRNP A1 or phnRNP A2. The bands representing unspliced HPV16 E6 mRNAs and HPV16 mRNAs spliced from SD226 to SA409 are indicated to the right. cDNAs were amplified by RT-PCR primers 97S and 438A. RT minus, RT-PCR performed in the absence or reverse transcriptase.

To more precisely map the target sequence of hnRNP A1 and A2, additional deletions were introduced in the 3′ end of plasmid pX856F, generating plasmids pX644, pX616, pX604, pX581, and pX579. The numerical digits in the plasmid names represent the endpoint of each deletion and refer to nucleotide position in the HPV16R sequence. As seen from the RT-PCR results obtained in cotransfections with empty pUC plasmid, splicing of mRNAs produced from pX556F, pX579, and pX581 was highly efficient, whereas splicing from plasmids pX604, pX614, and pX644 was less efficient (Fig. 6A and C). These results indicated that splicing inhibitory sequences extended to HPV16 nucleotide position 604 (Fig. 6A and C). RT-PCR bands were undetectable in control PCR experiments performed on the same RNA samples in the absence of reverse transcriptase (data not shown). Furthermore, similar results were obtained after transfection of the deletion mutants pX556F, pX579, pX604, pX614, and pX644 into HPV-negative cervical cancer cell line C33A (data not shown). Next, the same plasmids were transfected in the presence of phnRNP A1 plasmid. hnRNP A2 overexpression was not analyzed here, since HPV16 3′-splice site SA742 was absent from the reporter plasmids. As expected, overexpression of hnRNP A1 strongly inhibited splicing from the plasmids containing splicing inhibitory sequences (pX604, pX614, and pX644) and shifted splicing from a majority of spliced mRNAs (226̂409) to a majority of unspliced E6 mRNAs (Fig. 6A and B). In contrast, hnRNP A1 had no or very little effect on splicing of mRNAs produced from plasmids pX556F, pX579, and pX581 that did not contain splicing inhibitory sequences (Fig. 6A and B). These results suggested that hnRNPA1 acted on splicing inhibitory sequences located immediately upstream of HPV16 nucleotide position 604.

### The hnRNP A1 and A2 proteins target a UAG-encoding HPV16 sequence.

The sequence between HPV16 nucleotide positions 581 and 604 that inhibited splicing and that mediated the splicing-inhibitory effect of hnRNP A1 and hnRNP A2 encodes a UAG motif (Fig. 6C). Since hnRNP A1 has been shown to bind UAG-containing sequences, we introduced nucleotide substitutions in this sequence, resulting in plasmid pM604 (Fig. 6C). As can be seen, these point mutations inactivated the splicing silencer and enhanced splicing (Fig. 6D). Furthermore, the mutant plasmid pM604 responded less well to hnRNP A1 and hnRNP A2 overexpression than the wild-type plasmid pX604 (Fig. 6D), demonstrating that the UAG motif was required for splicing silencing and suggesting that hnRNP A1 and hnRNP A2 recognized this sequence.

### The hnRNP A1 and A2 proteins interact specifically with splicing inhibitory RNA sequences in the HPV16 E7 coding region.

**FIG 7:**
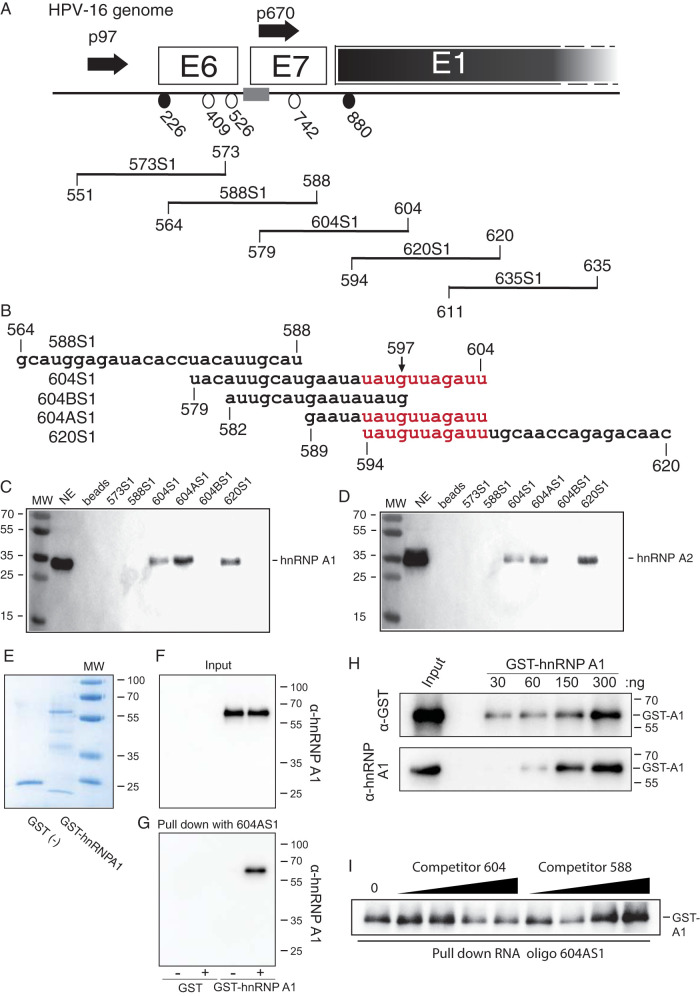
hnRNP A1 and A2 bind to an HPV16 RNA sequence located between HPV16 genomic nucleotide positions 594 and 604. (A) Schematic representation of the 5′ end of the HPV16 genome encoding E6, E7, and part of E1. HPV16 early (p97) and late (p670) promoters and HPV16 splice sites SD226, SA409, SA526, and SA742 are indicated. Names and nucleotide positions of overlapping, biotinylated RNA oligonucleotides are shown below. Nucleotide positions of the 5′ and 3′ ends of the RNA oligonucleotides refer to the HPV16 reference genome HPV16R (45). The region in the HPV16 genome covered by the RNA oligonucleotides is indicated as a gray box. (B) Sequences of selected biotinylated HPV16 RNA oligonucleotides used in RNA-mediated protein pulldowns of proteins from HeLa cell nuclear extracts. The sequence to which hnRNP A1 and A2 mapped is indicated in red. (C and D) Western blotting of factors pulled down by Streptavidin-coated magnetic beads carrying either of the indicated biotinylated HPV16 RNA oligonucleotides. Filters were stained with antibody to hnRNP A1 (C) or hnRNP A2 (D) as described in Materials and Methods. (E) Coomassie staining of purified GST- and GST-hnRNP A1 protein. (F) Western blotting of purified GST and GST-hnRNP A1 protein using anti-hnRNP A1 antibody. (G) Western blotting of GST-hnRNP A1 protein pulled down by Streptavidin-coated magnetic beads without RNA oligonucleotide or carrying biotinylated RNA oligonucleotide 604AS1. Filter was stained with antibody to hnRNP A1. (H) Serially diluted GST-hnRNP A1 was incubated with Streptavidin-coated magnetic beads bound to biotinylated RNA oligonucleotide 604S1 followed by magnetic pulldown of beads and Western blotting of hnRNP A1. (I) GST-hnRNP A1 was preincubated with nonbiotinylated RNA oligonucleotide 604 or 588 followed by incubation with Streptavidin-coated magnetic beads bound to biotinylated RNA oligonucleotide 604AS1. Pulled-down proteins were subjected to Western blotting with anti-hnRNP A1 antibody. MW, molecular weight markers; beads, pulldowns using beads only; NE, nuclear extract.

To determine if hnRNP A1 and A2 interact with the splicing inhibitory sequences immediately upstream of HPV16 nucleotide position 604, we performed RNA-mediated protein pulldowns using nuclear extract from HeLa cells and a set of nested, biotinylated HPV16 RNA oligonucleotides attached to Streptavidin-coated beads (Fig. 7A). The pulled-down factors were subjected to Western blotting with antibodies to hnRNP A1 or hnRNP A2. Only RNA oligonucleotides extending to HPV16 nucleotide position 604 pulled down hnRNAP A1, while oligonucleotides extending no further than to position 573 or 588 did not (Fig. 7C). Interestingly, hnRNP A2 interacted with the same RNA sequences (Fig. 7D). To more precisely map the target sequence for hnRNP A1 and A2, two shorter RNA oligonucleotides, named 604AS1 and 604BS1 (Fig. 7B), were used in the pulldown assay. Since hnRNP A1 and A2 were pulled down by 604AS1, the results presented in Fig. 7C and D collectively mapped the target site for both hnRNPA1 and A2 to sequences between HPV16 nucleotide positions 594 and 604. We concluded that hnRNP A1 and A2 interact with an 11-nucleotide, C-less sequence downstream of SA409 to inhibit utilization of this splice site. To determine if hnRNP A1 interacted directly with this sequence, glutathione *S*-transferase (GST) or GST-hnRNP A1 was used instead of nuclear extract in the pulldown assay (Fig. 7E and F). As can be seen, GST-hnRNP A1, but not GST, interacted with RNA oligonucleotide nucleotide 604AS1 (Fig. 7G). This interaction was GST-hnRNP A1 dose dependent (Fig. 7H). If GST-hnRNP A1 was preincubated with nonbiotinylated 604 RNA oligonucleotide, pulldown of GST-hnRNP A1 with the biotinylated 604AS1oligo was reduced (Fig. 7I), while preincubation with RNA oligonucleotide 588, in which the primary binding site of hnRNP A1 was absent, did not affect pulldown of GST-hnRNP A1 with biotinylated 604AS1oligo (Fig. 7I). Thus, hnRNP A1 interacted specifically and directly with the splicing inhibitory RNA sequence immediately upstream of HPV16 nucleotide position 604.

### The hnRNP A1 and A2 proteins interact with an UAG-encoding sequence.

**FIG 8:**
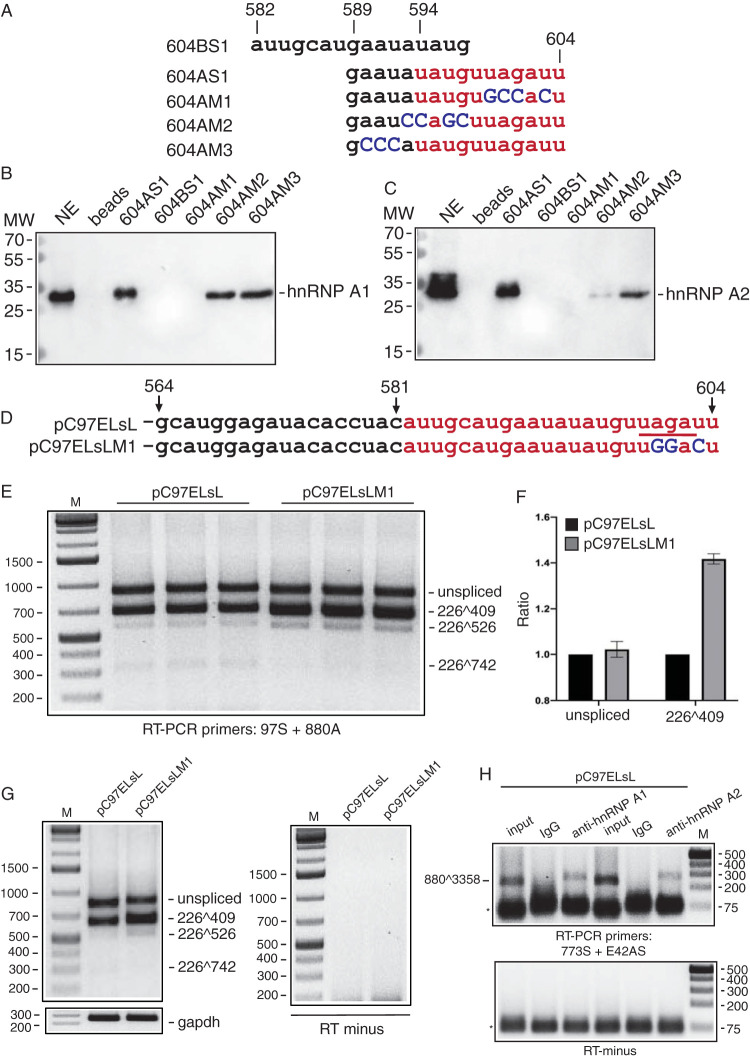
Nucleotide substitutions abolish pulldown of hnRNP A1 and A2 by HPV16 RNA oligonucleotides. (A) Sequences of biotinylated HPV16 RNA oligonucleotides used in RNA-mediated protein pulldowns of proteins from HeLa cell nuclear extracts. The sequence to which hnRNP A1 and A2 pulldown was mapped in Fig. 7 is indicated in red. Nucleotide substitutions are marked in dark blue. Numbers refer to nucleotide positions in HPV16R. Names of RNA oligonucleotides are listed to the left. (B and C) Western blotting of factors pulled down by Streptavidin-coated magnetic beads carrying either of the indicated biotinylated HPV16 RNA oligonucleotides. Filters were stained with antibody to either hnRNP A1 (B) or hnRNP A2 (C). (D) The sequence of the wild-type and mutant splicing silencer sequence in pC97ELsL and pC97ELsLM1, respectively, is shown. (E) RT-PCR on RNA extracted from HeLa cells transfected with plasmid pC97ELsL or pC97ELsLM1. RT-PCR primers 97S and 880A were used. (F) Quantitation of the triplicate samples. Mean values and standard deviations are shown. (G) RT-minus control. (H) HeLa cells were transfected with pC97ELsL in the presence of hnRNP A1 or hnRNP A2 as described in Materials and Methods. After 24 h, cells were lysed in RIP buffer (20 mM Tris-HCl, pH 8.0, 150 mM NaCl, 10% glycerol, 1% NP-40, 2 mM EDTA, pH 8.0, 400 U/ml Ribolock RNase inhibitor [ThermoFisher], protease inhibitor cocktail [Sigma]) and rotated for 1 h at 4°C. Samples were then centrifuged for 15 min at 14,000 × *g* at 4°C, and the supernatants were transferred to new tubes and incubated with 4 μg/ml anti-hnRNPA1 antibody (ab5832; Abcam), anti-hnRNPA2 antibody (ab227465; Abcam), or IgG overnight at 4°C. Each RNA-IP was incubated with 50 μl Dynabeads protein G (Life Technologies) for 1.5 h at 4°C, followed by washing four times in RIP buffer supplemented with 300 mM NaCl. The immunoprecipitated RNA then was extracted as described previously and subjected to RT-PCR by HPV16-specific primers 773s and E42AS, which detect HPV16 mRNA spliced between HPV16 splice sites SD880 and SA3358. Input represents RT-PCR with the same primers on RNA extracted from 5% of the input in the immunoprecipitation incubation. RT-minus, RT-PCR in the absence of reverse transcriptase. M, molecular weight marker; *, free primers.

Since we had shown in Fig. 6 that point mutations that changed the UAGAU sequence in HPV16 to GCCAC inactivated the splicing silencer in transient-transfection experiments, we introduced the same mutations in biotinylated RNA oligonucleotide 604AS1 (Fig. 8A), resulting in RNA oligonucleotide 604AM1 (Fig. 8A) (available on request). Two additional mutant oligonucleotides with substitutions immediately upstream of the UAGAU sequence, named 604AM2 and 604AM3 (Fig. 8A), were also utilized. As can be seen from the pulldown of proteins from HeLa nuclear extracts followed by Western blotting with antibodies to hnRNP A1 and hnRNP A2, mutations in the UAGAU sequence abolished hnRNP A1 and hnRNP A2 binding, whereas the upstream mutations did not (Fig. 8B and C). Similar results were obtained with both hnRNP A1 and hnRNP A2 (Fig. 8B and C), with the exception that mutations in 604AM2 immediately upstream of the UAGAU sequence reduced pulldown of hnRNP A1 but not hnRNP A2, suggesting that the hnRNP A1 binding site extends to the 5′ end of the UAGAU sequence. We concluded that nucleotide substitutions that abolished the splicing-inhibitory effect of the HPV16 splicing silencer in transfected cells also abolished pulldown *in vitro* of hnRNP A1 and hnRNP A2 by HPV16 RNA oligonucleotides.

In addition, we mutated the UAG-containing sequence in the context of the subgenomic HPV16 reporter plasmid pC97ELsL, resulting in pC97ELsLM1 (Fig. 8D). Analysis of RNA from HeLa cells transfected with pC97ELsL or pC97ELsLM1 revealed that pC97ELsLM1 produced more mRNA spliced from SD226 to SA409 than pC97ELsL (Fig. 8E and F). RT-PCR bands were not detected in the absence of reverse transcriptase (Fig. 8G). These results indicated that the UAG-containing splicing silencer is a major determinant of splicing to HPV16 SA409.

Finally, to confirm that hnRNP A1 and A2 interact with HPV16 early mRNAs in living cells, we immunoprecipitated RNA protein complexes from HeLa cells transfected with pC97ELsL in the presence of hnRNP A1 or A2 using monoclonal antibodies to either hnRNP A1 or hnRNP A2. RNA was extracted from the immunoprecipitations and subjected to RT-PCR with HPV16-specific PCR primers. As can be seen, monoclonal antibodies to hnRNP A1 or hnRNP A2 immunoprecipitated HPV16 mRNAs, whereas IgG did not (Fig. 8H). Thus, HPV16 early mRNAs interact with hnRNP A1 and hnRNP A2 in human cells.

### The C terminus of hnRNP A1 is required for efficient inhibition of HPV16 3′-splice site SA409 and for production of HPV16 unspliced E6 mRNAs.

**FIG 9:**
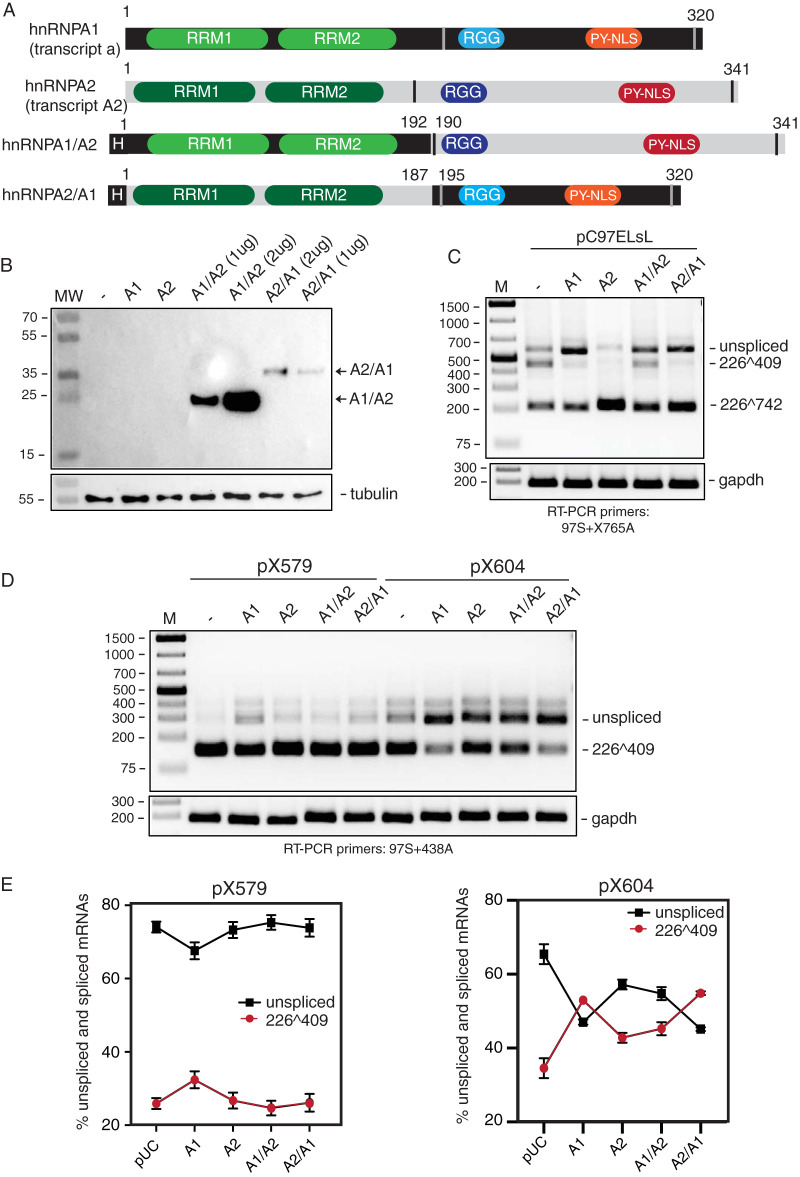
C terminus of hnRNP A1 is required for efficient inhibition of splicing between HPV16 5′-splice site SD226 and 3′-splice site SA409 in the E6 coding region. (A) Schematic representation of the hnRNP A1 and A2 proteins and the A1A2 and A2A1 hybrids. RNA recognition motifs 1 and 2 (RRM1 and RRM2), RGG, and pyrimidine-rich nuclear/cytoplasmic localization sequence (PY-NLS) (also termed M9) are indicated. hnRNP A1 consists of 320 amino acids and hnRNP A2 of 341 amino acids. The break points of the A1A2 and A2A1 hybrids are indicated. H, HA tag. (B) Western blotting on extracts from HeLa cells transfected with plasmids expressing the hnRNP A1A2 and hnRNP A2A1 hybrids. Blots are stained with anti-HA tag antibodies that detect the HA-tagged hnRNP A1A2 and hnRNP A2A1 hybrids but not the untagged hnRNP A1 and hnRNP A2 proteins. MW, molecular weight marker. (C) RT-PCR on RNA extracted from HeLa cells transfected with pC97ELsL (62) in the absence (−) or presence of phnRNP A1, phnRNP A2, or plasmids expressing the hnRNP A1A2 or hnRNP A2A1 hybrids (pA1NA2C and pA2NA1C). RT-PCR primers 97S and X765A were used (for the location of these RT-PCR primers, see Fig. 1C). The mRNA splicing each RT-PCR band represents is indicated to the right. (D) RT-PCR on RNA extracted from HeLa cells transfected with pX579 or pX604 in the absence (−) or presence of phnRNP A1 or phnRNP A2 plasmid or plasmids expressing the hnRNP A1A2 or hnRNP A2A1 hybrids. RT-PCR primers 97S and 438A were used. The splicing pattern each RT-PCR band represents is indicated to the right. (E) Densitometric quantification of RT-PCR bands shown in panel D. The quantitations are displayed as percent HPV16 unspliced mRNAs or percent HPV16 mRNAs spliced between SD226 and SA409 in each transfected sample. Quantitations were performed on triplicate samples. The left graph shows quantitations of RT-PCR bands obtained from transfections with HPV16 subgenomic reporter plasmid pX579, and the right graph shows those from transfections with pX604.

Both hnRNP A1 and A2 inhibit splicing between SD226 and SA409 and interact with the same splicing-inhibitory RNA element located between HPV16 nucleotide positions 594 and 604. Despite these similarities, overexpression of hnRNP A1 induced unspliced E6-encoding mRNAs, whereas overexpression of hnRNP A2 promoted E6̂E7-encoding mRNAs that were spliced between SD226 and SA742. hnRNP A1 and A2 are two closely related hnRNP proteins and display structural similarity with two conserved N-terminal RNA recognition motifs (RRMs), followed by a less conserved C-terminal glycine-rich domain that also harbors an RGG box and nuclear import and export signals. The N-terminal halves of the two proteins are relatively conserved, whereas the C-terminal part is more variable. To delineate various roles of hnRNP A1 and A2 protein domains in the control of HPV16 mRNA splicing, we generated hybrids between hnRNP A1 and A2 in which the two RRMs were derived from hnRNP A1 or A2 and the C terminus from either hnRNP A1 or A2, generating plasmids pA1NA2C and pA2NA1C (Fig. 9A). Western blotting confirmed that the HA-tagged hybrid proteins were expressed in HeLa cells (Fig. 9B). Next, the hybrids between hnRNP A1 and A2 were cotransfected with HPV16 reporter plasmid pC97ELsL, and RNA was extracted and analyzed by RT-PCR. Interestingly, the A1A2 hybrid, in which the two RRMs were derived from hnRNP A1 and the C terminus from hnRNP A2, inhibited splicing from SD226 to SA409 and promoted production of unspliced E6 mRNA, albeit with lower efficiency than wild-type hnRNP A1 (Fig. 9C). RT-PCR bands were undetectable in control PCR experiments performed on the same RNA samples in the absence of reverse transcriptase (data not shown). Thus, replacing the hnRNP A1 C terminus with the hnRNP A2 C terminus reduced efficiency of splicing inhibition, demonstrating an important role for the hnRNP A1 C terminus in splicing inhibition and production of HPV16 unspliced E6 mRNAs. On the other hand, the A2A1 hybrid, which contained the two RRMs from hnRNP A2 and the C terminus from hnRNP A1, inhibited splicing from SD226 to SA409 and promoted production of unspliced E6 mRNA in a manner that was similar to that of the wild-type hnRNP A1 (Fig. 9C), further corroborating the importance of the C terminus of hnRNP A1 in the generation of HPV16 unspliced E6 mRNAs. However, the A2A1 hybrid also induced splicing to SA742, although not as efficiently as wild-type hnRNP A2 (Fig. 9C). Thus, efficient splicing to SA742 required both N terminus and C terminus from hnRNP A2.

Since hnRNP A1 and A2 may act on multiple sites on HPV16 mRNAs, we next cotransfected the A1A2 and A2A1 hybrids with HPV16 reporter plasmid pX604 that contains only one target site for both hnRNP A1 and hnRNP A2. As expected, hnRNP A1 inhibited splicing between SD226 and SA409 more efficiently than hnRNP A2 (Fig. 9D and E), whereas no effect on pX579 was observed (Fig. 9D and E), as expected, since pX579 lacks the target site for hnRNP A1 and A2. Interestingly, the A2A1 hybrid inhibited splicing to the same extent as hnRNP A1 (Fig. 9D and E), whereas the ability of the A1A2 hybrid to inhibit splicing was lessened compared to that of wild-type hnRNP A1 (Fig. 9D and E). Combined, our results suggested that the conserved N terminus of hnRNP A1 and A2 interacted with the same HPV16 splicing inhibitory RNA element, and that the C terminus of hnRNP A1 was a more efficient inhibitor of splicing between HPV16 SD226 and SA409 than the C terminus of hnRNP A2. The results obtained with the hnRNP A1 and hnRNP A2 hybrids also strongly suggested that the activation of HPV16 SA742 by hnRNP A2 most likely was mediated by interactions of hnRNP A2 with sequences present elsewhere on the HPV16 pre-mRNAs than the splicing inhibitory between nucleotides 594 and 604. These other sequences were present in pC97ELsL but not in pX604. Taken together, hnRNP A1 and hnRNP A2 both inhibited splicing between the major HPV16 splice sites SD226 and SA409, but hnRNP A1 primarily promoted the production of unspliced E6-encoding mRNAs, while hnRNP A2 primarily promoted splicing to SA742, perhaps by interacting with other HPV16 sequences.

### Overexpression of hnRNP A1 or hnRNP A2 inhibits splicing between SD226 and SA409 on mRNAs produced from episomal HPV16 DNA.

**FIG 10:**
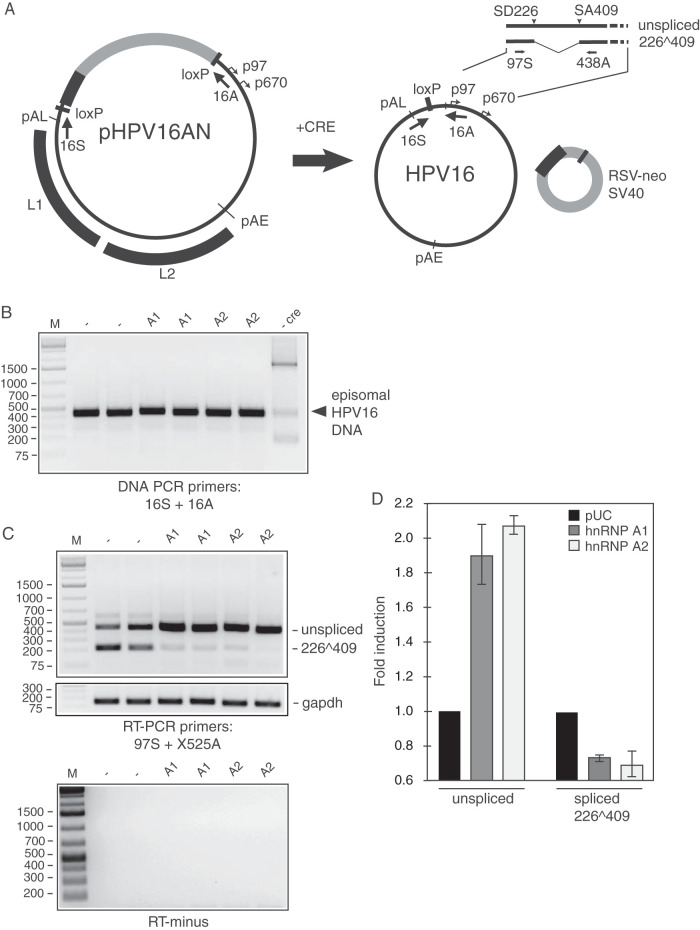
hnRNP A1 and A2 inhibit splicing of HPV16 E6 and E7 mRNAs produced from the episomal HPV16 DNA genome. (A, left) Schematic representation of genomic HPV16 plasmid pHPV16AN (40). LoxP sites and HPV16 early (p97) and late (p670) promoters and early (pAE) and late (pAL) poly(A) signals are indicated. Positions of PCR primers 16S and 16A are indicated. (Right) The effect of the cre recombinase on pHPV16AN in transfected cells is illustrated. Schematic representation of the 5′ end of the unspliced HPV16 E6 mRNAs and the mRNAs spliced between SD226 and SA409 are shown. (B) PCR with primers 16S and 16A on Hirt DNA extracted from HeLa cells transfected with the indicated HPV16 plasmids in the presence or absence (−) of the cre-expressing plasmid pCAGGS-nlscre. Primers 16S and 16A are located on each side of the LoxP sites in the HPV-16 plasmids, and the PCR reaction yields a 366-nucleotide PCR fragment that is diagnostic for recombination at the LoxP sites. A larger band is amplified from plasmid DNA that has not recombined (a parallel transfection performed in the absence of pCAGGS-nlscre [−cre]). (C) RT-PCR on RNA extracted from HeLa cells transfected with pHPV16AN and pCAGGS-nlscre in the absence (−) or presence of phnRNP A1 or phnRNP A2. Duplicate transfections are shown. RT-PCR primers 97S and X525A were used. The HPV16 splice sites used to generate the mRNAs amplified by the RT-PCR primers are indicated to the right. RT-minus, RT-PCR performed in the absence of reverse transcriptase. (D) Densitometric quantification of the RT-PCR bands on triplicates. Mean values are shown, and standard deviations are indicated. The quantitations are displayed as fold difference of HPV16 RT-PCR band intensity obtained with RNA extracted from cells transfected with pHPV16AN and pCAGGS-nlscre in the presence of phnRNP A1 or A2 over band intensities in cells transfected with pHPV16AN and pCAGGS-nlscre in the presence of empty pUC plasmid (−).

To investigate if overexpression of hnRNP A1 and A2 could inhibit splicing from SD226 to SA409 on HPV16 mRNAs produced from episomal genomic HPV16 DNA, we used plasmid pHPV16AN, which encodes the full-length HPV16 genome (Fig. 10A). The genomic plasmid pHPV16AN contains two lox sites that flank the HPV16 genome. Their presence allows the release and formation of the episomal form of the HPV16 genome by cotransfection with a cre enzyme-expressing plasmid (Fig. 10A). Plasmid pHPV16AN was cotransfected with a cre-expressing plasmid in the absence or presence of hnRNP A1 or A2 expression plasmid. Cre enzyme-mediated release and formation of circular episomal HPV16 DNA was confirmed by DNA extraction and DNA PCR using primers 16S and 16A flanking the loxP sites (Fig. 10B). As can be seen, the episomal form of the HPV16 genome was efficiently produced in the presence of the cre enzyme (Fig. 10B). Overexpression of hnRNP A1 or A2 inhibited HPV16 mRNA splicing and promoted production of unspliced E6 mRNAs over HPV16 mRNAs spliced between SD226 and SA409 (Fig. 10C). Quantitation of the RT-PCR bands is shown in Fig. 10D. We concluded that overexpression of hnRNP A1 or A2 inhibited splicing between HPV16 SD226 and SA409 on mRNAs produced from the episomal form of the HPV16 genome.

### Knockdown of hnRNP A1 enhances splicing between HPV16 SD226 and SA409 on mRNAs produced from episomal HPV16 genome.

**FIG 11:**
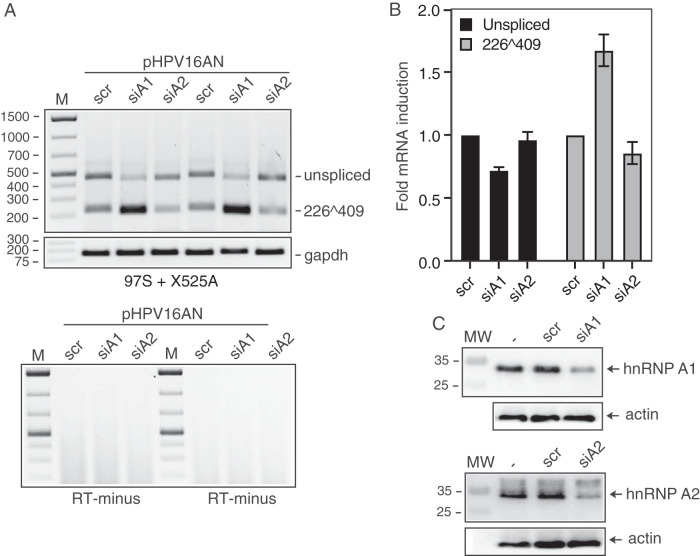
Knockdown of hnRNP A1 enhances splicing of HPV16 E6 and E7 mRNAs produced from the episomal HPV16 DNA genome. (A) pHPV16AN (40) and pCAGGS-nlscre were cotransfected in duplicates with scrambled (scr) siRNAs or siRNAs to hnRNP A1 (siA1) or hnRNP A2 (siA2), RNA was extracted, and RT-PCR was performed with primers 97S and X525A. Control PCRs in the absence of reverse transcriptase (RT-minus) are shown. (B) Densitometric quantification of the RT-PCR bands was performed in triplicates. Mean values are shown and standard deviations are indicated. The quantitations are displayed as fold difference of HPV16 RT-PCR band intensity obtained with RNA extracted from cells transfected with pHPV16AN and pCAGGS-nlscre in the presence of hnRNP A1 or hnRNP A2 siRNAs over band intensities in cells transfected with pHPV16AN and pCAGGS-nlscre in the presence of scrambled siRNAs (scr). (C) Knockdown of hnRNP A1 and hnRNP A2 was confirmed by Western blotting of hnRNP A1 or hnRNP A2.

Since overexpression of hnRNP A1 or hnRNP A2 inhibited splicing between SD226 and SA409, knockdown of hnRNP A1 or hnRNP A2 should have the opposite effect. Transfection of full-length HPV16 genome pHPV16AN+cre with scrambled short interfering RNAs (siRNAs) or siRNAs directed to either hnRNP A1 or hnRNP A2, revealed that knockdown of hnRNP A1 resulted in enhanced splicing between SD226 and SA409 (Fig. 11A and B), whereas knockdown of hnRNP A2 had no measurable effect on splicing between SD226 and SA409 (Fig. 11A and B). Western blotting revealed that the siRNAs specifically reduced levels of hnRNP A1 or hnRNP A2 protein in the siRNA-transfected cells (Fig. 11C). Taken together, these results confirmed that hnRNP A1 controlled HPV16 E6 and E7 mRNA splicing and supported the idea that high levels of hnRNP A1 inhibit splicing between SD226 and SA409, thereby promoting production of full-length HPV16 E6 mRNA.

### Knockdown of hnRNP A1 in HPV16-positive cervical cancer cell line SiHa alters HPV16 E6/E7 mRNA splicing and increases HPV16 E7 protein levels.

**FIG 12:**
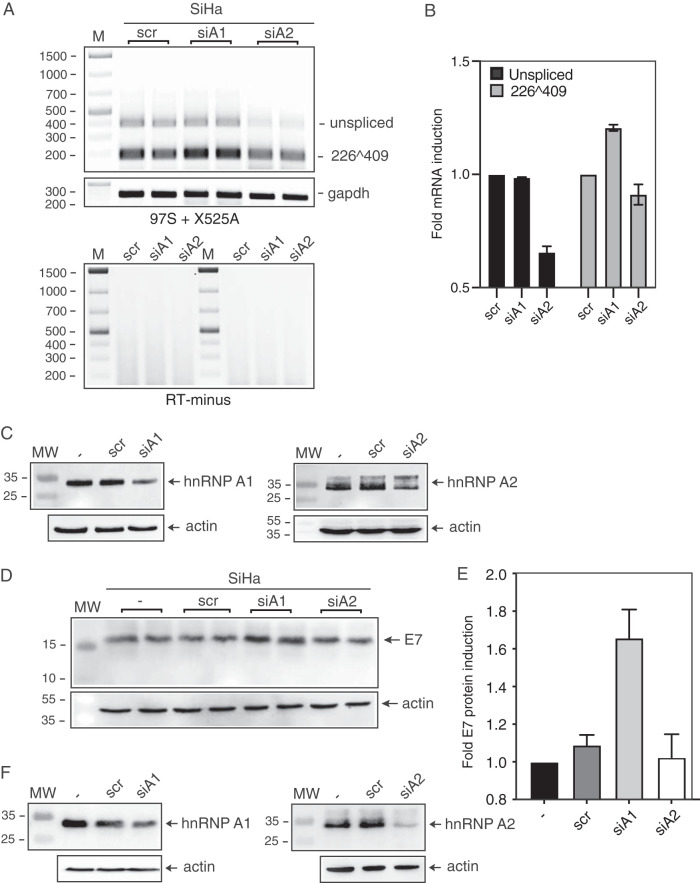
Knockdown of hnRNP A1 in HPV16-driven cervical cancer cell line SiHa promotes splicing of the HPV16 E6 and E7 mRNAs and enhances production of HPV16 E7 protein. (A) HPV16-positive cervical cancer cell line SiHa was transfected in duplicates with scrambled (scr) siRNAs or siRNAs to hnRNP A1 (siA1) or hnRNP A2 (siA2), RNA was extracted, and RT-PCR was performed with primers 97S and X525A. (B) Densitometric quantification of the RT-PCR bands was performed in triplicates. Mean values are shown, and standard deviations are indicated. The quantitations are displayed as fold difference of HPV16 RT-PCR band intensity obtained with RNA extracted from SiHa cells transfected with siRNAs to hnRNP A1 or hnRNP A2 (siA1 and siA2) siRNAs over band intensities in cells transfected with scrambled siRNAs (scr). (C) Knockdown of hnRNP A1 and hnRNP A2 was confirmed by Western blotting of hnRNP A1 or hnRNP A2. (D) HPV16-positive cervical cancer cell line SiHa was either untransfected (−) or transfected in duplicates with scrambled (scr) siRNAs or siRNAs to hnRNP A1 (siA1) or hnRNP A2 (siA2), and protein was extracted with RIPA buffer and analyzed by Western blotting with antibodies to HPV16 E7 or actin. (E) Densitometric quantification of the HPV16 E7 bands was performed on triplicates. Mean values are shown and standard deviations are indicated. The quantitations are displayed as fold difference of HPV16 E7 protein band intensity obtained with proteins extracted from SiHa cells transfected with hnRNP A1 siRNAs (siA1) or with hnRNP A2 siRNAs (siA2) over band intensities in cells transfected with scrambled siRNAs (scr). (F) Knockdown of hnRNP A1 and hnRNP A2 was confirmed by Western blotting of hnRNP A1 or hnRNP A2.

To investigate if knockdown of hnRNP A1 or A2 affected splicing of HPV16 mRNAs in HPV16-driven cancer cells, HPV16-positive cervical cancer cell line SiHa was transfected with siRNAs to hnRNP A1 or hnRNP A2. As can be seen, knockdown of hnRNP A1 enhanced levels of the HPV16 E7 mRNAs spliced between SD226 and SA409 (Fig. 12B and C), whereas knockdown of hnRNP A2 caused a reduction of unspliced E6 mRNAs without substantially affecting HPV16 mRNAs spliced between SD226 and SA409 (Fig. 12B and C). As predicted, knockdown of hnRNP A1 also enhanced production of the HPV16 E7 protein, whereas knockdown of hnRNP A2 had no monitorable effect on E7 (Fig. 12D, E, and F). We concluded that hnRNP A1 contributed to the control of HPV16 E7 protein production in HPV16-positive cancer cells by regulating HPV16 mRNA splicing.

## DISCUSSION

hnRNP A1 is a modular RNA binding protein consisting of two RNA recognition motifs (RRM1 and RRM2) and a glycine-rich C-terminal region rich in Arg-Gly-Gly (RGG) motifs that regulates alternative splicing and contributes to mRNA nuclear export, translation, and other RNA processing events (46). hnRNP A1 functions as an inhibitor of RNA splicing in many, if not all, cases by promoting exon skipping (47–49). In contrast, in HPV16, hnRNP A1 promotes retention of an intron that also encodes a portion of the E6 protein. Thus, splicing inhibition and intron retention by hnRNP A1 in HPV16 generates the only HPV16 mRNA with ability to produce full-length E6 protein. The mechanism by which hnRNP A1 causes intron retention in HPV16 is unknown but may be similar to the exon-skipping mechanism, in which it has been proposed that initial binding of hnRNP A1 to a high-affinity binding site promotes cooperative binding of hnRNP A1 to the same mRNA, thereby extending hnRNP A1 RNA binding to splicing enhancers nearby and blocking their function (46). Alternatively, hnRNP A1 and splicing-enhancing factors are antagonists that compete for the same or overlapping binding sites. It remains to be determined how hnRNP A1 promotes HPV16 E6 intron retention.

The retained intron in the HPV16 E6 coding region is located between HPV16 splice sites SD226 and SA409. When 5′-splice site SD226 is not used, the HPV16 mRNAs will contain at least one unutilized 5′-splice site (SD226) and at least three unutilized 3′-splice sites (SA409, SA526, and SA742). Such splice sites may cause nuclear retention of these mRNAs, as the mRNAs are deemed immature and not ready for further processing and export to the cytoplasm. In that regard, it is interesting that hnRNP A1 has a shuttling function and potentially aids in the nuclear export of the HPV16 E6 mRNAs with retained introns. The PY-NSL sequence (also called M9) in the glycine-rich region of hnRNP A1 interacts with transportin 1 and 2 and appears to act as a bidirectional nuclear-cytoplasmic shuttling signal, probably with the help of transportin, as no export-specific partner of hnRNP A1 has been identified.

hnRNP A1 binding sites have been determined previously by SELEX (5′-UAGGGA/U-3′) (50) as well as by transcriptome-wide studies (5′-UAG-3′) (51, 52). Both RRM1 and RRM2 can bind RNA simultaneously and with similar sequence specificity and affinity. The preferred binding site for RRM1 was shown to be 5′-U/CAGG-3′, and that for RRM2 was 5′-U/CAGN-3′ (53). Binding of hnRNP A1 to these sequences was particularly sensitive to C-substitutions, particularly AG to CG (54, 55). In line with these previous observations, we mapped the hnRNP A1 binding site in HPV16 to the 5′-UAUGUUAGAUU-3′ sequence in the HPV16 E7 coding region, downstream of 3′-splice site SA409. This is an 11-nucleotide, C-less sequence with a UAG motif in the middle, just like many of the previously mapped hnRNP A1-binding sites and splicing regulatory RNA elements (Fig. 7).

**FIG 13:**
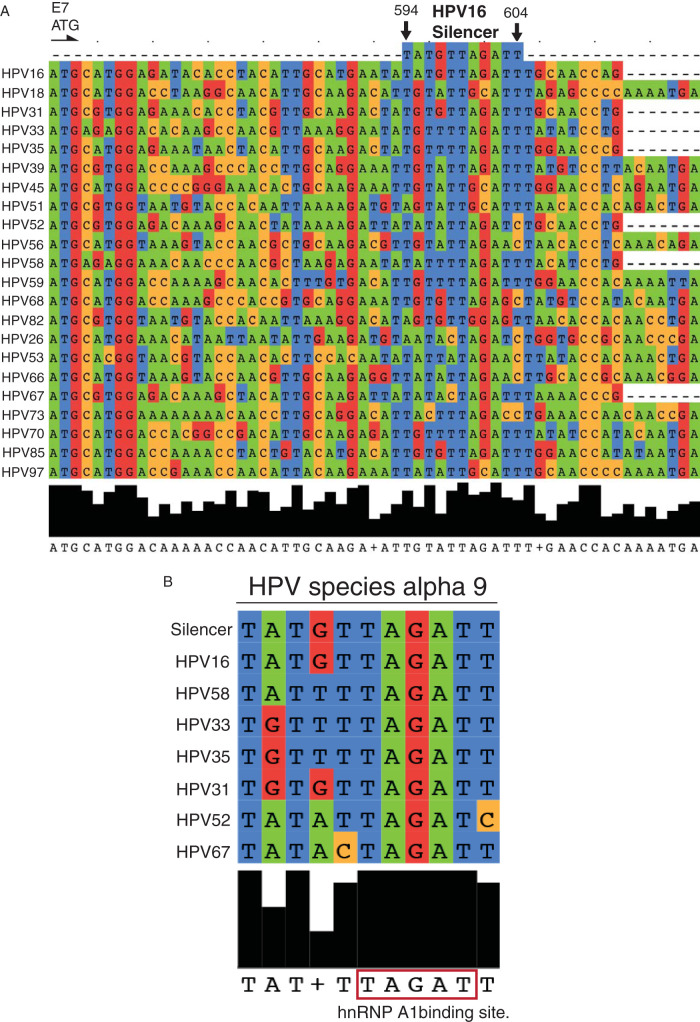
(A) Sequence alignment of high-risk HPV nucleotide sequences spanning the HPV16 silencer element (see Materials and Methods). The E7 ATG and HPV16 nucleotide positions 594 and 606, which flank the HPV16 splicing silencer element in HPV16, are indicated. (B) Strict conservation of the hnRNP A1 binding site in HPV-16 containing HPV species alpha 9. Alignments are in Jalview 2.0.5 with Muscle of HPV sequences from HPV species alpha-9.

The HPV16 3′-splice site SA409 in the E6 coding region is a hallmark of the high-risk, cancer-associated HPV types and is a very efficiently utilized splice site. It generates the HPV16 E7 mRNA by shortening the upstream E6 orf by splicing to create an mRNA that is efficiently translated into E7 protein (26, 36). One can speculate that this splice site is of significance, primarily since it generates the E7 mRNAs of the high-risk HPV types, although it has been suggested that this mRNA produces a shorter protein, named E6*I, that has a distinct function (32). Interestingly, the regulatory RNA element identified here (5′-UAUGUUAGAUU-3′) is located in the E7 coding region, even though it controls splice site SA409, which is located in the E6 coding region. According to previous reports, the E7 sequence is particularly well conserved, even compared with E6 (56). Therefore, we performed a sequence alignment of the 11-nucleotide RNA element and hnRNP A1 binding site that we had identified. Even though the sequence is not well conserved among all high-risk types (Fig. 13A), the UAGAU sequence that appears to be of key importance for hnRNP A1 binding is strictly conserved in all high-risk HPV types in the HPV alpha-9 species (Fig. 13B). Interestingly, this sequence is only moderately conserved in the HPV alpha-7 species that includes HPV18, despite the fact that it has been shown that hnRNP A1 inhibits the splice sites in the HPV18 E6 region. The binding site for hnRNP A1 in HPV18 (39) was mapped to sequences upstream and downstream of the hnRNP A1 site identified here in HPV16. Since hnRNP A1 now has been shown to inhibit splicing in the E6 region of both HPV16 and HPV18, it is reasonable to speculate that hnRNP A1 plays a significant role in the control of HPV16 and HPV18 E6 and E7 expression. hnRNP A1 has a splicing inhibitory role and promotes production of unspliced E6-encoding mRNAs while inhibiting E7 mRNA and protein production. However, it is the spliced mRNA that produces E7 protein that is the most common of the E6 and E7 mRNAs (28, 29, 31). It has also been suggested that splicing factor SRSF2/SC35 stabilizes all alternatively spliced HPV16 E6 mRNAs and the E7 mRNA (57). Even if hnRNP A1 is required for upholding the levels of unspliced E6 mRNAs, another cellular factor must operate in high-risk HPV16-infected cells to promote efficient splicing to 3′-splice site SA409 in the E6 region. Alternatively, hnRNP A1 or other cellular factors contribute to control of HPV16 E6/E7 mRNA splicing by interacting with the branch point or with SF1, which recognizes the branch point located upstream of the 3′-splice sites (33). One can speculate that knockdown or overexpression of hnRNP A1 and/or A2 indirectly affects HPV16 mRNA splicing by altering expression of other cellular RNA binding proteins, such as SR proteins, that contribute to control of HPV16 mRNAs splicing. Further experiments are required to understand how expression of high-risk E6 and E7 expression is controlled by alternative splicing.

The HPV16 E7 protein is the major driver of cell proliferation in HPV16-infected cells, including infected keratinocytes and HPV16-driven cancer cells (9). As unscheduled cell proliferation activated by E7 also induces apoptosis, inhibition of cell apoptosis by HPV16 E6 protein is of paramount importance (10). Since HPV16 E6 and E7 are produced from two mutually exclusive, alternatively spliced mRNAs, a balanced splicing is critical for survival of the HPV16-infected cells. This simultaneous production and relative levels of E6 and E7 are controlled by the ratio of unspliced E6 mRNAs to spliced E7 mRNAs. Cancer cells driven by HPV16 would succumb to apoptosis or would enter a state of senescence if either E6 or E7 levels were altered. Thus, splicing factors that control the ratio of the alternatively spliced HPV16 E6 and E7 mRNAs play a key role in HPV16-caused cancer. Cellular factors that control this splicing event are of major importance not only in the HPV16 replication cycle but also during carcinogenesis. It was previously shown that the levels of RNA binding proteins in HPV16-infected cervical epithelium change during carcinogenesis as well as in response to cell differentiation (22, 23). We have shown that the levels of hnRNP A1 increased in premalignant lesions as well as in malignant cervical lesions and cervical cancer (22). In other cancer cells, it has been shown that knockdown of hnRNP A1 induces apoptosis (58), suggesting a general role for hnRNP A1 in maintaining the cancer phenotype of a transformed cell (59). Taken together, these observations support an important role for hnRNP A1 in the HV16 life cycle as well as during HPV16-induced carcinogenesis. However, it is the spliced form of the mRNA that encodes E7 that dominates in HPV16 infected cells. The cellular factor that promotes this splicing event and antagonizes hnRNP A1 remains to be identified.

If hnRNP A1 plays an important role in determining the optimal ratio of HPV16 E6 and E7 mRNAs and proteins, it is important to control the activity of hnRNP A1. This protein can be phosphorylated, sumoylated, and acetylated (46). A previous study suggested that splicing in the HPV16 E6 region is controlled by the epidermal growth factor pathway through hnRNP A1 (38). It was shown that activation of the Erk1/2-kinase pathway promoted retention of the E6 intron and production of E6 mRNA. Knockdown of hnRNP A1, but not hnRNP A2, prevented Erk1/2-induced intron retention in the E6 region, which is in line with the results presented here, and suggested that hnRNP A1-mediated splicing inhibition and intron retention is controlled by phosphorylation of hnRNP A1. Phosphorylation of cellular splicing factors has also been shown to play a role in HPV16 late gene expression, as the Akt-kinase phosphorylates hnRNP L to suppress HPV16 late gene expression (60). The Akt-kinase also appears to play a role in the control SR proteins that regulate bovine papillomavirus late gene expression (61). One can speculate that inhibitors of enzymes that posttranslationally modify RNA binding proteins that control HPV16 gene expression by splicing can be used for treatment of premalignant lesions or cancer caused by HPV16.

Finally, we found that hnRNP A1 and A2 have opposite effects on this very vulnerable HPV16 splicing event in the E6 coding region, despite the fact that hnRNP A1 and A2 are two of the most closely related proteins in the hnRNP family of RNA-binding proteins. From a mechanistic point of view, it would be interesting to determine how these two proteins affect splicing of HPV16 E6 and E7 mRNAs in such a diametrically opposed manner. Our results suggest that the reason for the differential effects by hnRNP A1 and A2 on HPV16 mRNA splicing is primarily caused by differences in the less conserved C-terminal portion of the two proteins. Our results also suggested that hnRNP A2 interacts with other, downstream RNA sequences to promote splicing to the downstream 3′-splice SA742 rather than causing intron retention in the E6 coding region. Further experiments are needed to determine how hnRNP A2 promotes splicing to HPV16 SA742, a splice site required for production of the E6̂E7 fusion protein of unknown function and for the E1 and E1̂E4 mRNAs expressed from the HPV16 early promoter p97.

## MATERIALS AND METHODS

### Plasmids.

The following plasmids were described previously: pC97ELsL (62) and pHPV16AN (40). Plasmids phnRNP A1 and phnRNP A2 express the hnRNP A1 or A2 cDNAs from the CMV promoter.

Primer B97S was used in combination with primer X1200A, X1060A, X960A, X856AF, X644A, X616A, X604A, M604A, X581A, X579A, or X556AF to amplify HPV16 sequences that were digested with PteI and XhoI and inserted between the PteI and XhoI sites in plasmid pC0806 (63), thereby generating CMV promoter-driven plasmids pX1200, pX1060, pX960, pX856F, pX644, pX616, pX604, pM604A pX581, pX579, and pX556F. All plasmids end at HPV16 nucleotide positions indicated in the plasmid names, e.g., p1200 ends at HPV16 genomic nucleotide position 1200. All HPV16 subgenomic expression plasmids contain the HPV16 late polyadenylation signal pAL.

To generate plasmid pA1NA2C, we PCR amplified the N terminus of hnRNP A1 with primers HAA1S and A1PSTEA, cut the PCR fragment with SalI and PteI, and inserted it into plasmid pBEL cut with the same enzymes. The resulting intermediate plasmid was digested with BssHII and XhoI and ligated to a PCR fragment generated from hnRNP A2 with primers A2PSTES and A2XA and cut with the same enzymes. To generate plasmid pA2NA1C, we PCR amplified the N terminus of hnRNP A2 with primers HAA2S and A2PSTEA, cut the PCR fragment with SalI and PteI, and inserted it into plasmid pBEL (42) cut with the same enzymes. The resulting intermediate plasmid was digested with PteI and XhoI and ligated to a PCR fragment generated from hnRNP A1 with primers A1PSTES and A1XA and cut with the same enzymes. Sequences of the PCR primers used for plasmid constructions are available on request.

### Cells.

HeLa cells and HPV16-positive SiHa cells were cultured in Dulbecco’s modified Eagle medium (GE Healthcare Life Science HyClone Laboratories) with 10% bovine calf serum (GE Healthcare Life Science HyClone Laboratories) and 1% penicillin-streptomycin (Gibco Thermo Fisher Science).

### Transfections.

Transfections of HeLa cells were carried out using TurboFect according to the manufacturer’s instructions (Thermo Fisher Science). TurboFect was mixed with plasmid DNA and incubated at room temperature for 15 min prior to dropwise addition to 60-mm plates with subconfluent HeLa cells. Cells were harvested at 20 h posttransfection. Each plasmid was transfected in triplicate in a minimum of two independent experiments. For analysis of episomal HPV16, plasmid pHPV16AN (40) was cotransfected with plasmid pCAGGS-nlscre (generously provided by Andras Nagy at University of Toronto), which expresses the cre recombinase that releases the HPV16 genome from the plasmid at two flanking lox sites.

### siRNA transfection.

siRNA knockdowns were carried out using DharmaFECT transfection reagents according to the manufacturer's instructions. Briefly, the siRNA was diluted to 40 nM final concentration in 250 μl serum-free medium, and the mixture was added to 250 μl of serum-free medium with 5 μl transfection reagent. The mixture was incubated at room temperature for 20 min prior to addition to a 60-mm plate with subconfluent HeLa or SiHa cells. siRNA to hnRNPA1 was ON-TARGET plus SMART pool human hnRNPA1 (L-008221-00-0020; Dharmacon) and to hnRNPA2B1 ON-TARGET plus SMART pool human hnRNPA2B1 (L-011690-01-0020; Dharmacon). The scrambled control (scr) was siGENOME control pool nontargeting number 2 (D-001206-14-20; Dharmacon).

### RNA extraction and RT-PCR.

Total RNA was extracted using TRI Reagent and a Direct-zol RNA MiniPrep kit (ZYMO Research) according to the manufacturer’s protocol. One microgram of total RNA was reverse transcribed in a 20-μl reaction mixture at 37°C by using M-MLV reverse transcriptase (Invitrogen) and random primers (Invitrogen) according to the protocol of the manufacturer. One microliter of cDNA was subjected to PCR amplification. HPV16 mRNAs spliced from HPV16 5′-splice site SD226 to 3′-splice site SA409 were amplified with RT-PCR primers 97S and 438A and from HPV16 5′-splice site SD226 to 3′-splice sites SA409, SA526, and SA742 with RT-PCR primers 97S and 880A. HPV16 mRNAs spliced from 5′-splice site SD880 to 3′-splice site SA2709 or SA3358 were amplified with RT-PCR primers 773S and E2A or E4A. HPV16 late L1 mRNAs were amplified with RT-PCR primers 773S and L1A. Glyceraldehyde-3-phosphate dehydrogenase (GAPDH) cDNA was amplified with primers GAPDH-F and GAPDH-R. To monitor recombination at the loxP sites in pHPV16AN, PCR was performed with primers 16S and 16A on DNA extracted from the transfected cells (this PCR yields a 366-nucleotide PCR fragment that is diagnostic for recombination at the LoxP sites). Sequences of the RT-PCR primers are available on request. Examples of control PCR experiments performed on RNA samples in the absence of reverse transcriptase are shown in the various figures.

### Protein extraction and Western immunoblotting.

Proteins for Western blotting were extracted from cells using the radioimmunoprecipitation assay (RIPA) buffer (50 mM Tris, pH 7.4, 500 mM NaCl, 1% Na-DOC, 0,1% SDS, 1% Triton X-100) with 30 min of incubation on ice and occasional vortexing. Western blotting was performed as described previously (60). The following antibodies were used: anti-hnRNP A1 (04-1469; Millipore), anti-hnRNP A2B1 (ab227465; Abcam), anti-HPV16 E7 (GTX133411; GeneTex), anti-beta tubulin (T9026; Sigma-Aldrich), anti-actin (SC-1616; Santa Cruz), anti-HA tag (SC7392; Santa Cruz), and anti-GST (A5800; Invitrogen) antibody. Secondary antibodies conjugated with horseradish peroxidase were used, and proteins were detected using the Clarity Western ECL substrate (Bio-Rad) or the Super Signal West Femto chemiluminescence substrate (Pierce).

### ssRNA-mediated protein pulldown assay.

Nuclear extracts were prepared according to the procedure described previously (60). Briefly, the cells were lysed using lysis buffer A (10 mM HEPES, 1.5 mM MgCl_2_, 10 mM KCl, 0.5 mM dithiothreitol, 0.05% NP-40, pH 7.9, and protease inhibitors) to release cytoplasmic proteins. The pelleted nuclei were resuspended in buffer B (5 mM HEPES, 1.5 mM MgCl_2_, 0.2 mM EDTA, 0.5 mM dithiothreitol, 26% glycerol, pH 7.9, and protease inhibitor) to release nuclear proteins. The nuclear extracts were mixed with streptavidin-coated magnetic beads (Dynabeads M-280 Streptavidin; Invitrogen) bound to biotin-labeled single-stranded RNA (ssRNA) oligonucleotides (Sigma-Aldrich) in binding buffer (10 mM Tris, pH 7.4, 150 mM NaCl, 2.5 mM MgCl_2_, 0.5% Triton X-100). Sequences of all biotinylated ssRNA oligonucleotides are available on request. The mixtures were incubated at room temperature with rotation for 1 h, followed by washing five times with 1 ml wash buffer. Proteins were eluted by boiling of the beads in SDS-PAGE loading buffer and subjected to SDS-PAGE followed by Western blotting with the indicated antibodies. GST and GST-hnRNP A1 were purified from Escherichia coli transformed with pGEX-derived plasmids using GS beads.

### Quantitations.

The software used to determine band intensity in Western blots and RT-PCR gels was Image Lab 6.0.1, and quantitations were performed with the software Prism GraphPad 8.4.0.

### Sequence alignment.

The software for alignments was Jalview 2.0.5, and the alignment method was Muscle.

## References

[1] Chow LT, Broker TR, Steinberg BM 2010 The natural history of human papillomavirus infections of the mucosal epithelia. APMIS 118:422–449. doi:10.1111/j.1600-0463.2010.02625.x. 20553526

[2] Schiffman M, Doorbar J, Wentzensen N, de Sanjose S, Fakhry C, Monk BJ, Stanley MA, Franceschi S 2016 Carcinogenic human papillomavirus infection. Nat Rev Dis Primers 2:16086. doi:10.1038/nrdp.2016.86. 27905473

[3] Zur Hausen H 2002 Papillomaviruses and cancer: from basic studies to clinical application. Nat Rev Cancer 2:342–350. doi:10.1038/nrc798. 12044010

[4] Walboomers JMM, Jacobs MV, Manos MM, Bosch FX, Kummer JA, Shah KV, Snijders PJF, Peto J, Meijer CJLM, Muñoz N 1999 Human papillomavirus is a necessary cause of invasive cervical cancer worldwide. J Pathol 189:12–19. doi:10.1002/(SICI)1096-9896(199909)189:1<12::AID-PATH431>3.0.CO;2-F. 10451482

[5] Kajitani N, Satsuka A, Kawate A, Sakai H 2012 Productive lifecycle of human papillomaviruses that depends upon squamous epithelial differentiation. Front Microbiol 3:152. doi:10.3389/fmicb.2012.00152. 22536200 PMC3334820

[6] Hong S, Laimins LA 2013 Regulation of the life cycle of HPVs by differentiation and the DNA damage response. Future Microbiol 8:1547–1557. doi:10.2217/fmb.13.127. 24266355 PMC3951404

[7] Doorbar J, Quint W, Banks L, Bravo IG, Stoler M, Broker TR, Stanley MA 2012 The biology and life-cycle of human papillomaviruses. Vaccine 30(Suppl 5):F55–F70. doi:10.1016/j.vaccine.2012.06.083. 23199966

[8] Mighty KK, Laimins LA 2014 The role of human papillomaviruses in oncogenesis. Recent Results Cancer Res 193:135–148. doi:10.1007/978-3-642-38965-8_8. 24008297

[9] Roman A, Munger K 2013 The papillomavirus E7 proteins. Virology 445:138–168. doi:10.1016/j.virol.2013.04.013. 23731972 PMC3783579

[10] Vande Pol SB, Klingelhutz AJ 2013 Papillomavirus E6 oncoproteins. Virology 445:115–137. doi:10.1016/j.virol.2013.04.026. 23711382 PMC3783570

[11] Thierry F 2009 Transcriptional regulation of the papillomavirus oncogenes by cellular and viral transcription factors in cervical carcinoma. Virology 384:375–379. doi:10.1016/j.virol.2008.11.014. 19064276

[12] Bernard HU 2013 Regulatory elements in the viral genome. Virology 445:197–204. doi:10.1016/j.virol.2013.04.035. 23725692

[13] McBride AA 2013 The papillomavirus E2 proteins. Virology 445:57–79. doi:10.1016/j.virol.2013.06.006. 23849793 PMC3783563

[14] Johansson C, Schwartz S 2013 Regulation of human papillomavirus gene expression by splicing and polyadenylation. Nat Rev Microbiol 11:239–251. doi:10.1038/nrmicro2984. 23474685

[15] Schwartz S 2013 Papillomavirus transcripts and posttranscriptional regulation. Virology 445:187–196. doi:10.1016/j.virol.2013.04.034. 23706315

[16] Jia R, Zheng ZM 2009 Regulation of bovine papillomavirus type 1 gene expression by RNA processing. Front Biosci 14:1270–1282. doi:10.2741/3307. PMC2654602 19273129

[17] Graham SV, Faizo AA 2017 Control of human papillomavirus gene expression by alternative splicing. Virus Res 231:83–95. doi:10.1016/j.virusres.2016.11.016. 27867028 PMC5335905

[18] Wu C, Kajitani N, Schwartz S 2017 Splicing and polyadenylation of human papillomavirus type 16 mRNAs. Int J Mol Sci 18:366. doi:10.3390/ijms18020366. PMC5343901 28208770

[19] Zheng ZM, He P, Baker CC 1996 Selection of the bovine papillomavirus type 1 nucleotide 3225 3′ splice site is regulated through an exonic splicing enhancer and its juxtaposed exonic splicing suppressor. J Virol 70:4691–4699. doi:10.1128/JVI.70.7.4691-4699.1996. 8676495 PMC190405

[20] Zheng ZM, He PJ, Baker CC 1997 Structural, functional, and protein binding analyses of bovine papillomavirus type 1 exonic splicing enhancers. J Virol 71:9096–9107. doi:10.1128/JVI.71.12.9096-9107.1997. 9371566 PMC230210

[21] Zheng ZM, He PJ, Baker CC 1999 Function of a bovine papillomavirus type 1 exonic splicing suppressor requires a suboptimal upstream 3′ splice site. J Virol 73:29–36. doi:10.1128/JVI.73.1.29-36.1999. 9847303 PMC103804

[22] Fay J, Kelehan P, Lambkin H, Schwartz S 2009 Increased expression of cellular RNA-binding proteins in HPV-induced neoplasia and cervical cancer. J Med Virol 81:897–907. doi:10.1002/jmv.21406. 19319956

[23] Mole S, McFarlane M, Chuen-Im T, Milligan SG, Millan D, Graham SV 2009 RNA splicing factors regulated by HPV16 during cervical tumour progression. J Pathol 219:383–391. doi:10.1002/path.2608. 19718710 PMC2779514

[24] Nilsson K, Wu C, Kajitani N, Yu H, Tsimtsirakis E, Gong L, Winquist EB, Glahder J, Ekblad L, Wennerberg J, Schwartz S 2018 The DNA damage response activates HPV16 late gene expression at the level of RNA processing. Nucleic Acids Res 46:5029–5049. doi:10.1093/nar/gky227. 29596642 PMC6007495

[25] Nilsson K, Wu C, Schwartz S 2018 Role of the DNA damage response in human papillomavirus RNA splicing and polyadenylation. Int J Mol Sci 19:1735. doi:10.3390/ijms19061735. PMC6032147 29895741

[26] Tang S, Tao M, McCoy JP, Zheng Z-M 2006 The E7 oncoprotein is translated from spliced E6*I transcripts in high-risk human papillomavirus type 16- or 18-positive cervical cancer cell lines via translation reinitiation. J Virol 80:4249–4263. doi:10.1128/JVI.80.9.4249-4263.2006. 16611884 PMC1472016

[27] Zheng ZM, Tao M, Yamanegi K, Bodaghi S, Xiao W 2004 Splicing of a cap-proximal human papillomavirus 16 E6E7 intron promotes E7 expression, but can be restrained by distance of the intron from its RNA 5′ cap. J Mol Biol 337:1091–1108. doi:10.1016/j.jmb.2004.02.023. 15046980

[28] Schmitt M, Dalstein V, Waterboer T, Clavel C, Gissman L, Pawlita M 2010 Diagnosing cervical cancer and high-grade precursors by HPV-16 transcription patterns. Cancer Res 70:249–256. doi:10.1158/0008-5472.CAN-09-2514. 20028865

[29] Schmitt M, Pawlita M 2011 The HPV transcriptome in HPV16 positive cells. Mol Cell Probes 25:108–113. doi:10.1016/j.mcp.2011.03.003. 21439369

[30] Smotkin D, Prokoph H, Wettstein FO 1989 Oncogenic and nononcogenic human genital papillomaviruses generate the E7 mRNA by different mechanisms. J Virol 63:1441–1447. doi:10.1128/JVI.63.3.1441-1447.1989. 2536845 PMC247848

[31] Cornelissen MT, Smits HL, Briët MA, van den Tweel JG, Struyk AP, van der Noordaa J, ter Schegget J 1990 Uniformity of the splicing pattern of the E6/E7 transcripts in human papillomavirus type 16-transformed human fibroblasts, human cervical premalignant lesions and carcinomas. J Gen Virol 71:1243–1246. doi:10.1099/0022-1317-71-5-1243. 2161056

[32] Olmedo-Nieva L, Munoz-Bello JO, Contreras-Paredes A, Lizano M 2018 The role of E6 spliced isoforms (E6*) in human papillomavirus-induced carcinogenesis. Viruses 10:45. doi:10.3390/v10010045. PMC5795458 29346309

[33] Ajiro M, Jia R, Zhang L, Liu X, Zheng ZM 2012 Intron definition and a branch site adenosine at nt 385 control RNA splicing of HPV16 E6*I and E7 expression. PLoS One 7:e46412. doi:10.1371/journal.pone.0046412. 23056301 PMC3464268

[34] Brant AC, Majerciak V, Moreira MAM, Zheng ZM 2019 HPV18 utilizes two alternative branch sites for E6*I splicing to produce E7 protein. Virol Sin 34:211–221. doi:10.1007/s12250-019-00098-0. 30945125 PMC6513837

[35] Remm M, Remm A, Ustav M 1999 Human papillomavirus type 18 E1 protein is translated from polycistronic mRNA by a discontinuous scanning mechanism. J Virol 73:3062–3070. doi:10.1128/JVI.73.4.3062-3070.1999. 10074156 PMC104066

[36] Stacey SN, Jordan D, Snijders PJF, Mackett M, Walboomers JMM, Arrand JR 1995 Translation of the human papillomavirus type 16 E7 oncoprotein from bicistronic mRNA is independent of splicing events within the E6 open reading frame. J Virol 69:7023–7031. doi:10.1128/JVI.69.11.7023-7031.1995. 7474122 PMC189622

[37] del Moral-Hernández O, López-Urrutia E, Bonilla-Moreno R, Martínez-Salazar M, Arechaga-Ocampo E, Berumen J, Villegas-Sepúlveda N 2010 The HPV-16 E7 oncoprotein is expressed mainly from the unspliced E6/E7 transcript in cervical carcinoma C33-A cells. Arch Virol 155:1959–1970. doi:10.1007/s00705-010-0787-9. 20865289

[38] Rosenberger S, De-Castro Arce J, Langbein L, Steenbergen RDM, Rösl F 2010 Alternative splicing of human papillomavirus type-16 E6/E6* early mRNA is coupled to EGF signaling via Erk1/2 activation. Proc Natl Acad Sci U S A 107:7006–7011. doi:10.1073/pnas.1002620107. 20351270 PMC2872467

[39] Ajiro M, Tang S, Doorbar J, Zheng ZM 2016 Serine/arginine-rich splicing factor 3 and heterogeneous nuclear ribonucleoprotein A1 regulate alternative RNA splicing and gene expression of human papillomavirus 18 through two functionally distinguishable cis elements. J Virol 90:9138–9152. doi:10.1128/JVI.00965-16. 27489271 PMC5044842

[40] Li X, Johansson C, Glahder J, Mossberg AK, Schwartz S 2013 Suppression of HPV-16 late L1 5′-splice site SD3632 by binding of hnRNP D proteins and hnRNP A2/B1 to upstream AUAGUA RNA motifs. Nucleic Acids Res 41:10488–10508. doi:10.1093/nar/gkt803. 24013563 PMC3905901

[41] Zhao X, Fay J, Lambkin H, Schwartz S 2007 Identification of a 17-nucleotide splicing enhancer in HPV-16 L1 that counteracts the effect of multiple hnRNP A1-binding splicing silencers. Virology 369:351–363. doi:10.1016/j.virol.2007.08.002. 17869320

[42] Zhao X, Rush M, Schwartz S 2004 Identification of an hnRNP A1 dependent splicing silencer in the HPV-16 L1 coding region that prevents premature expression of the late L1 gene. J Virol 78:10888–10905. doi:10.1128/JVI.78.20.10888-10905.2004. 15452209 PMC521837

[43] Zhao X, Schwartz S 2008 Inhibition of HPV-16 L1 expression from L1 cDNAs correlates with the presence of hnRNP A1 binding sites in the L1 coding region. Virus Genes 36:45–53. doi:10.1007/s11262-007-0174-0. 18040766

[44] Orru B, Cunniffe C, Ryan F, Schwartz S 2012 Development and validation of a novel reporter assay for human papillomavirus type 16 late gene expression. J Virol Methods 183:106–116. doi:10.1016/j.jviromet.2012.03.023. 22484615

[45] Van Doorslaer K, Tan Q, Xirasagar S, Bandaru S, Gopalan V, Mohamoud Y, Huyen Y, McBride AA 2013 The Papillomavirus Episteme: a central resource for papillomavirus sequence data and analysis. Nucleic Acids Res 41:D571–D578. doi:10.1093/nar/gks984. 23093593 PMC3531071

[46] Jean-Philippe J, Paz S, Caputi M 2013 hnRNP A1: the Swiss army knife of gene expression. Int J Mol Sci 14:18999–19024. doi:10.3390/ijms140918999. 24065100 PMC3794818

[47] Mayeda A, Krainer AR 1992 Regulation of alternative pre-mRNA splicing by hnRNP A1 and splicing factor SF2. Cell 68:365–375. doi:10.1016/0092-8674(92)90477-t. 1531115

[48] Caceres JF, Stamm S, Helfman DM, Krainer AR 1994 Regulation of alternative splicing in vivo by overexpression of antagonistic splicing factors. Science 265:1706–1709. doi:10.1126/science.8085156. 8085156

[49] Yang X, Bani MR, Lu SJ, Rowan S, Ben-David Y, Chabot B 1994 The A1 and A1B proteins of heterogeneous nuclear ribonucleoparticles modulate 5′ splice site selection in vivo. Proc Natl Acad Sci U S A 91:6924–6928. doi:10.1073/pnas.91.15.6924. 8041722 PMC44310

[50] Burd CG, Dreyfuss G 1994 RNA binding specificity of hnRNP A1: significance of hnRNP A1 high-affinity binding sites in pre-mRNA splicing. EMBO J 13:1197–1204. doi:10.1002/j.1460-2075.1994.tb06369.x. 7510636 PMC394929

[51] Bruun GH, Doktor TK, Borch-Jensen J, Masuda A, Krainer AR, Ohno K, Andresen BS 2016 Global identification of hnRNP A1 binding sites for SSO-based splicing modulation. BMC Biol 14:54. doi:10.1186/s12915-016-0279-9. 27380775 PMC4932749

[52] Huelga SC, Vu AQ, Arnold JD, Liang TY, Liu PP, Yan BY, Donohue JP, Shiue L, Hoon S, Brenner S, Ares M Jr, Yeo GW 2012 Integrative genome-wide analysis reveals cooperative regulation of alternative splicing by hnRNP proteins. Cell Rep 1:167–178. doi:10.1016/j.celrep.2012.02.001. 22574288 PMC3345519

[53] Beusch I, Barraud P, Moursy A, Clery A, Allain FH 2017 Tandem hnRNP A1 RNA recognition motifs act in concert to repress the splicing of survival motor neuron exon 7. Elife 6:e25736. doi:10.7554/eLife.25736. 28650318 PMC5503513

[54] Rollins C, Levengood JD, Rife BD, Salemi M, Tolbert BS 2014 Thermodynamic and phylogenetic insights into hnRNP A1 recognition of the HIV-1 exon splicing silencer 3 element. Biochemistry 53:2172–2184. doi:10.1021/bi500180p. 24628426 PMC3985463

[55] Tavanez JP, Madl T, Kooshapur H, Sattler M, Valcarcel J 2012 hnRNP A1 proofreads 3′ splice site recognition by U2AF. Mol Cell 45:314–329. doi:10.1016/j.molcel.2011.11.033. 22325350

[56] Mirabello L, Yeager M, Yu K, Clifford GM, Xiao Y, Zhu B, Cullen M, Boland JF, Wentzensen N, Nelson CW, Raine-Bennett T, Chen Z, Bass S, Song L, Yang Q, Steinberg M, Burdett L, Dean M, Roberson D, Mitchell J, Lorey T, Franceschi S, Castle PE, Walker J, Zuna R, Kreimer AR, Beachler DC, Hildesheim A, Gonzalez P, Porras C, Burk RD, Schiffman M 2017 HPV16 E7 genetic conservation is critical to carcinogenesis. Cell 170:1164–1174. doi:10.1016/j.cell.2017.08.001. 28886384 PMC5674785

[57] McFarlane M, MacDonald AI, Stevenson A, Graham SV 2015 Human papillomavirus 16 oncoprotein expression is controlled by the cellular splicing factor SRSF2 (SC35). J Virol 89:5276–5287. doi:10.1128/JVI.03434-14. 25717103 PMC4442513

[58] Patry C, Bouchard L, Labrecque P, Gendron D, Lemieux B, Toutant J, Lapointe E, Wellinger R, Chabot B 2003 Small interfering RNA-mediated reduction in heterogeneous nuclear ribonucleoparticule A1/A2 proteins induces apoptosis in human cancer cells but not in normal mortal cell lines. Cancer Res 63:7679–7688. 14633690

[59] Roy R, Huang Y, Seckl MJ, Pardo OE 2017 Emerging roles of hnRNPA1 in modulating malignant transformation. Wiley Interdiscip Rev RNA 8:e1431. doi:10.1002/wrna.1431. 28791797

[60] Kajitani N, Glahder J, Wu C, Yu H, Nilsson K, Schwartz S 2017 hnRNP L controls HPV16 RNA polyadenylation and splicing in an Akt-kinase-dependent manner. Nucleic Acids Res 45:9654–9678. doi:10.1093/nar/gkx606. 28934469 PMC5766200

[61] Liu X, Mayeda A, Tao M, Zheng ZM 2003 Exonic splicing enhancer-dependent selection of the bovine papillomavirus type 1 nucleotide 3225 3′ splice site can be rescued in a cell lacking splicing factor ASF/SF2 through activation of the phosphatidylinositol 3-kinase/Akt pathway. J Virol 77:2105–2115. doi:10.1128/jvi.77.3.2105-2115.2003. 12525645 PMC140879

[62] Li X, Johansson C, Cardoso-Palacios C, Mossberg A, Dhanjal S, Bergvall M, Schwartz S 2013 Eight nucleotide substitutions inhibit splicing to HPV-16 3′-splice site SA3358 and reduce the efficiency by which HPV-16 increases the life span of primary human keratinocytes. PLoS One 8:e72776. doi:10.1371/journal.pone.0072776. 24039800 PMC3767658

[63] Collier B, Öberg D, Zhao X, Schwartz S 2002 Specific inactivation of inhibitory sequences in the 5′ end of the human papillomavirus type 16 L1 open reading frame results in production of high levels of L1 protein in human epithelial cells. J Virol 76:2739–2752. doi:10.1128/jvi.76.6.2739-2752.2002. 11861841 PMC135970

